# Bioengineering of injectable encapsulated aggregates of pluripotent stem cells for therapy of myocardial infarction

**DOI:** 10.1038/ncomms13306

**Published:** 2016-10-27

**Authors:** Shuting Zhao, Zhaobin Xu, Hai Wang, Benjamin E. Reese, Liubov V. Gushchina, Meng Jiang, Pranay Agarwal, Jiangsheng Xu, Mingjun Zhang, Rulong Shen, Zhenguo Liu, Noah Weisleder, Xiaoming He

**Affiliations:** 1 Department of Biomedical Engineering, The Ohio State University, Columbus, Ohio 43210, USA; 2 Davis Heart and Lung Research Institute, The Ohio State University, Columbus, Ohio 43210, USA; 3 Department of Physiology and Cell Biology, The Ohio State University, Columbus, Ohio 43210, USA; 4 Division of Cardiology, Department of Medicine, The Ohio State University, Columbus, Ohio 43210, USA; 5 Department of Pathology, The Ohio State University, Columbus, Ohio 43210, USA; 6 Comprehensive Cancer Center, The Ohio State University, Columbus, Ohio 43210, USA

## Abstract

It is difficult to achieve minimally invasive injectable cell delivery while maintaining high cell retention and animal survival for *in vivo* stem cell therapy of myocardial infarction. Here we show that pluripotent stem cell aggregates pre-differentiated into the early cardiac lineage and encapsulated in a biocompatible and biodegradable micromatrix, are suitable for injectable delivery. This method significantly improves the survival of the injected cells by more than six-fold compared with the conventional practice of injecting single cells, and effectively prevents teratoma formation. Moreover, this method significantly enhances cardiac function and survival of animals after myocardial infarction, as a result of a localized immunosuppression effect of the micromatrix and the *in situ* cardiac regeneration by the injected cells.

## Abstract (web summary)

Stem cell therapy of myocardial infarction is hampered by poor survival of injected cells. Here the authors develop injectable aggregates of stem cells differentiated to an early cardiac stage and encapsulated in a biodegradable micromatrix, and show their enhanced therapeutic efficacy in a heart infarction mouse model.

Myocardial infarction (MI) is a leading cause of death globally^1^,^2^. This is due, in part, to the fact that the human heart has a very limited capacity of self-repair, and that there is no clinical treatment targeting the loss of cardiomyocytes (CMs) following MI (refs 3, 4, 5). Stem cell therapy (SCT) has been explored as a promising option for regenerating cardiac tissue, including CMs, to treat MI. Various types of stem cells have been investigated exhibiting both advantages and disadvantages. To date, only pluripotent stem cells (PSCs), including embryonic stem cells (ESCs) and induced pluripotent stem cells (iPSCs), are well accepted to be capable of differentiating into functional CMs^3^,^4^,^5^,^6^,^7^. However, the delivery of stem cells needs significant further improvement regardless of which types of stem cells are used.

The retention of single (that is, dissociated) stem cells in the infarct zone delivered in suspension has been dismal (often <∼10% within a few hours to a few days post injection)^5^,^8^,^9^,^10^. Delivery of stem cells in tissue-engineered constructs in the form of a macro-scale (up to a few centimeters) hydrogel, porous scaffold, or cell sheet/patch may improve cell retention. However, there is significant cell death inside the macro-scale constructs due to the limited diffusion length of oxygen *in vivo* (<∼150 μm), and it may require multiple surgeries to overcome the diffusion limit of oxygen *in vivo* for using cell sheets/patches <∼150 μm thick as 1D microscale stem cell constructs^9^,^10^.

In addition, the retained cells may die of the hostile MI microenvironment that could be exacerbated by the implanted cells to trigger immune reactions^11^,^12^,^13^,^14^. The presence of macrophages together with the cytokines secreted by them in the first few days after MI, creates a strong pro-inflammatory environment resulting in chemo-attraction of more immune cells and damage to the transplanted stem cells^15^,^16^. Therefore, injection of stem cells at 4–7 days after MI may help to improve the survival of the implanted/retained cells^16^,^17^. However, significant injury to the infarcted myocardium would accumulate during the 4–7 days of delay. Therefore, early treatment to minimize the injury or pathological development after MI is desired. Temporary systemic immunosuppression for a few days has been proposed to mitigate immune rejection to the implanted stem cells to improve their survival^11^,^12^,^13^. However, systemic immunosuppression could induce severe complications to patients including infection and possible cancer occurence^18^,^19^.

Lastly, it has been reported that surviving PSCs may form teratomas, consisting of cells of all the three different lineages (that is, ectoderm, mesoderm and endoderm), in the heart^8^,^20^,^21^,^22^,^23^,^24^,^25^. To overcome this concern, PSCs have been differentiated into mature cardiomyocytes before implantation to minimize the risk of teratoma formation^24^,^25^. However, implantation of mature cardiomyocytes has been reported to cause an electromechanical mismatch with the host cardiomyocytes^26^. Therefore, it might be advantageous to pre-differentiate the PSCs into the early cardiac stage rather than into mature cardiomyocytes for implantation into the heart. This approach would then utilize the native chemical, mechanical and electrical cues in the heart to further guide the pre-differentiated cells (at the early cardiac stage) into mature cardiomyocytes with similar electromechanical properties to the native CMs.

To address the aforementioned challenges, we report an effective approach to prepare PSCs for implantation to treat MI in this study. This approach is inspired by the multi-step natural procedure of preparing totipotent-pluripotent cells for implantation into the uterus wall in the female reproductive system, including their proliferation, pre-differentiation, re-encapsulation, hatching and eventually implantation. This approach may be valuable to facilitate the clinical application of SCT for treating MI and possibly many other degenerative diseases.

## Results

### Preparing PSCs for implantation by injection to treat MI

**Figure 1:**
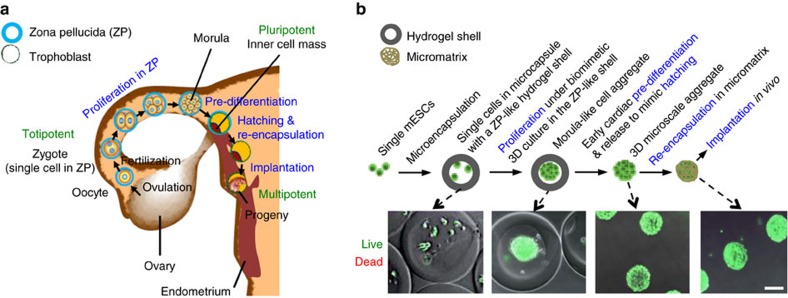
Bioinspired approach for preparing pluripotent stem cells to implant by injectable delivery. (**a**) A schematic illustration of the multi-step procedure to prepare the totipotent-pluripotent stem cells for implantation in the uterus wall, including proliferation to form a microscale cell aggregate (that is, morula) in zona pellucida, pre-differentiation of morula into trophoblast cells and inner cell mass in the zona pellucida, hatching out of the zona pellucida and re-encapsulation in the trophoblast before implantation during early embryo development in the female reproductive system. (**b**) A schematic illustration of the bioinspired procedure for producing 3D microscale constructs of murine embryonic stem cells (mESCs) together with real images, showing the analogy between the bioinspired approach and the aforementioned natural procedure. The bioinspired approach mimics the natural procedure phenomenologically rather than mechanistically. Scale bar, 100 μm

Our approach for preparing PSCs for implantation by injectable delivery to treat MI is inspired by the multi-step procedure used by nature to prepare totipotent-pluripotent stem cells for implantation into the uterus. This natural procedure includes the following steps (Fig. 1a)^27^,^28^,^29^: proliferation of the totipotent-pluripotent stem cells in the miniaturized permissive core enclosed in the semipermeable shell (known as zona pellucida) of pre-hatching embryos into a multicellular aggregate known as morula, pre-differentiation of the aggregated cells into trophoblast cells and inner cell mass, hatching out of zona pellucida and re-encapsulation within the trophoblast, and implantation into the uterus wall. Because our experimental strategy shares some similarity with the basic steps of the early embryonic development and includes proliferation, pre-differentiation, zona hatching and re-encapsulation of the natural procedure, we dubbed it the ‘bioinspired' approach. (illustrated in Fig. 1b). First, we microencapsulated ∼21±4 single (that is, dissociated) murine ESCs (mESCs) in the permissive liquid core of microcapsules with a semipermeable alginate hydrogel shell produced using the coaxial electrospray technology^29^ (Supplementary Fig. 1) for culture. The mESCs proliferate to form one single aggregate of 128.9±17.4 μm consisting of 1,450±381 cells per aggregate with high (>90%) viability in each microcapsule in 7 days. The core–shell architecture of the microcapsules mimics the physical configuration of pre-hatching embryo with a hydrogel-like shell (that is, zona pellucida) and a permissive core, the native home of stem cells from the totipotent to pluripotent stage^27^,^28^,^29^. After forming the mESC aggregates, we pre-differentiated them into the early cardiac stage inside the microcapsules. Afterward, the pre-differentiated aggregates were released from the core–shell microcapsules by dissolving the alginate hydrogel shell using an isotonic solution of sodium citrate and further re-encapsulated within a micromatrix for implantation. This does not significantly affect their size and integrity (see the images in Fig. 1b) and enables injectable delivery (intramyocardial injection) of the pre-differentiated aggregates as a result of their miniaturized dimension. More data on characterizing the early cardiac pre-differentiation of the mESC aggregates and re-encapsulation of the pre-differentiated aggregates within a micromatrix are given below.

### Pre-differentiating mESC aggregates to early cardiac stage

**Figure 2:**
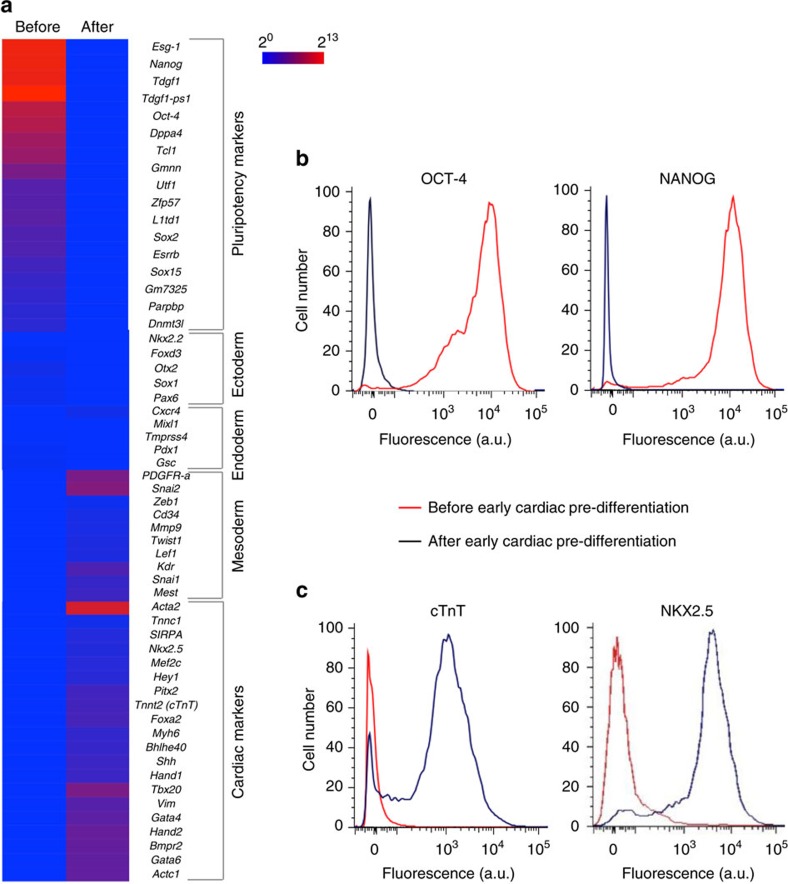
Pre-differentiation of the mESC aggregates into the early cardiac stage. (**a**) Microarray data showing significantly increased expression of mesoderm and cardiac marker genes and significantly decreased expression of pluripotency marker genes in the aggregated cells after pre-differentiation. (**b**) Flow cytometry data showing successful pre-differentiation of the mESC aggregates with diminished expression of pluripotency protein makers (OCT-4 and NANOG). (**c**) Flow cytometry data showing early cardiac pre-differentiation with significantly increased expression of cardiac specific protein marker (cTnT) and the early cardiac protein marker (NKX2.5).

To minimize the issue of teratoma associated with using PSCs in their pluripotent state for tissue regeneration^20^,^21^, we pre-differentiated the mESC aggregates for 3 days before implantation using BMP-4 and bFGF to specifically direct the cells towards an early cardiac stage^30^,^31^. Successful pre-differentiation is confirmed by RNA microarray data (Fig. 2a) showing that the expression of cardiac marker genes was significantly higher in the pre-differentiated mESCs than the cells before pre-differentiation^30^,^31^,^32^,^33^. The expression of other typical genes that regulate heart and mesoderm development were also up-regulated, while the pluripotency marker genes were down-regulated in the pre-differentiated cells. We validated the microarray analysis with the conventional method of analysing gene expression using quantitative reverse transcription polymerase chain reaction (qRT-PCR) to quantify the expression of the early cardiac gene marker *Nkx*2.5 (refs 30, 31, 32, 33). Both the microarray and qRT-PCR analyses show that the expression of the *Nkx*2.5 gene was significantly up-regulated by 4–5 times after pre-differentiation (Fig. 2a; Supplementary Fig. 2). Further flow cytometry analyses were conducted for typical pluripotency protein markers (OCT-4 and NANOG, Fig. 2b), and cardiac specific protein makers including cardiac troponin T (cTnT) and NKX2.5 (Fig. 2c)^4^,^6^,^8^,^31^,^32^,^33^. These altogether with immunohistostaining analysis (Supplementary Fig. 3a,b) of the early cardiac protein marker (NKX2.5) confirmed that the aggregated cells were successfully directed into the early cardiac lineage after pre-differentiation. This is because the pluripotency markers in the cells after pre-differentiation are negligible, while the expression of cardiac specific markers (particularly the early cardiac marker) is significantly increased after the early cardiac pre-differentiation treatment.

### Re-encapsulating the pre-differentiated aggregates

**Figure 3:**
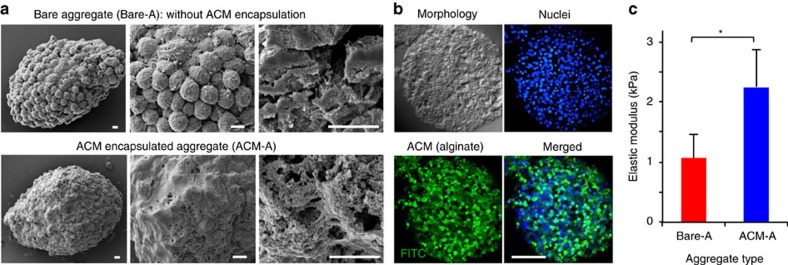
Characterization of the aggregates pre-differentiated to the early cardiac stage. (**a**) SEM images showing successful encapsulation of the pre-differentiated cell aggregates with the alginate-chitosan micromatrix (ACM) both outside (first two columns) and inside (third column) the aggregates. Scale bars, 5 μm. (**b**) Confocal fluorescence micrographs of the middle plane of the ACM-A showing micromatrix inside the aggregates indicated by labelling alginate in the ACM with FITC to show up with green fluorescence. Scale bar, 50 μm. (**c**) The elastic modulus of the pre-differentiated aggregates is significantly increased after ACM encapsulation. The modulus was determined by atomic force microscopy (AFM) nanoindentation. Error bars represent standard deviation (s.d., *n*=3). *P<0.05 (Student two-tailed *t-*test).

After the early cardiac pre-differentiation, we released the aggregates from the core–shell microcapsules using isotonic sodium citrate solution^34^,^35^. We further re-encapsulated the released aggregates in an alginate-chitosan micromatrix (ACM) by soaking the aggregates first in chitosan (0.4% w/v in saline), and then in a solution (0.15% w/v in saline) of oxidized alginate^34^,^36^. The oxidized alginate degrades much faster than the regular alginate in the core–shell microcapsules^36^. This soaking procedure was repeated once. As shown in the first two columns of Fig. 3a, individual cells on the surface of bare pre-differentiated aggregate (Bare-A, top) are clearly visible, while the aggregate with ACM encapsulation (ACM-A, bottom) is covered with materials. Moreover, as shown in the third column of Fig. 3a for cut-open aggregates, an extensive micromatrix of materials that cover the cells is visible inside the ACM-A (bottom), while it is minimal with visible individual cells inside the Bare-A (top). The micromatrix inside the ACM-A is visualized by confocal fluorescence microscopy (Fig. 3b; Supplementary Movie 1) showing green fluorescence throughout the ACM-A, due to labelling the alginate in the ACM (only for this experiment) with a green fluorescent probe fluorescein isothiocyanate (FITC). Furthermore, the elastic modulus of ACM-A determined by atomic force microscopy (AFM) nanoindentation is significantly higher than that of Bare-A (Fig. 3c).

**Figure 4:**
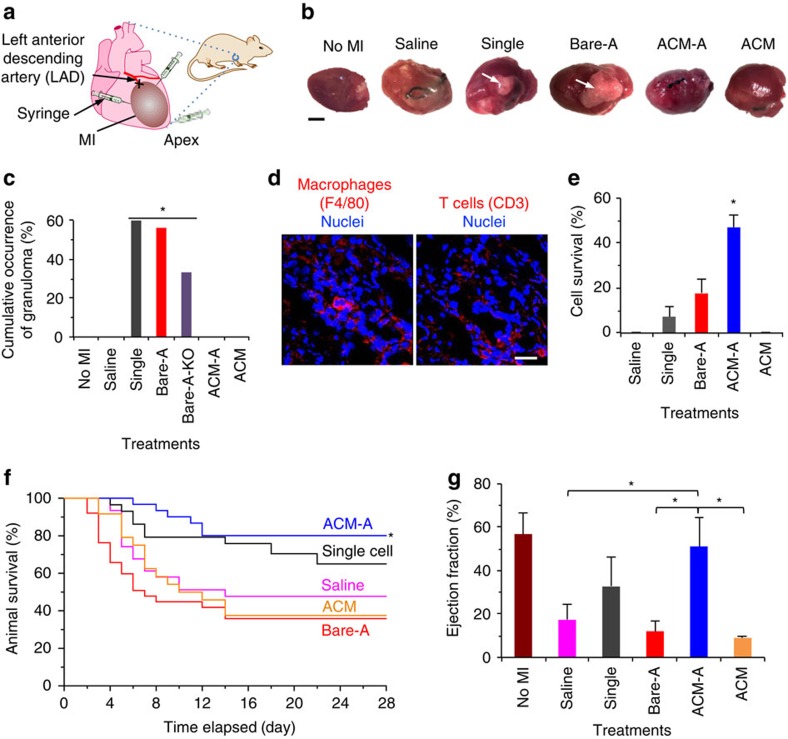
Therapy of myocardial infarction by injecting ACM-encapsulated pre-differentiated aggregates. (**a**) A schematic illustration of surgical ligation (X) of the LAD at its proximal location to create large-area myocardial infarction (MI) and implantation of samples by intramyocardial injection at three different locations. (**b**) Typical gross images of a heart with no MI and MI hearts with five different treatments showing granulomas in single cell (Single) and Bare-A treated mice (arrows). Scale bar, 3 mm. (**c**) Quantitative data of cumulative granuloma occurrence in both wild-type (WT) and *Card9* knockout (KO) mice, showing treatments with single, Bare-A, Bare-A-KO have significantly higher occurrence of granuloma than the other treatments including ACM-A. The animal number (n) was 10, 31, 29, 38, 31, 24 and 9 for No MI, Saline, Single, Bare-A, ACM-A, ACM and Bare-A-KO, respectively. *P<0.05 (Chi-square test). (**d**) Typical micrograph of granuloma with immunofluorescence staining of F4/80 (for macrophage, red) and CD3 (for T cells, red) showing many immune cells within a loose matrix in the granuloma collected at 28 days after injected with Bare-A. The nuclei are stained blue. Scale bar, 20 μm. (**e**) Data of implanted cells (expressing green fluorescence protein, GFP) retained and survived in the heart after 28 days showing a significantly higher cell survival with the ACM-A treatment than all the other treatments. The cell survival was quantified by counting cells with green fluorescence in the heart from the apex to the point of ligation. Error bars represent s.d. (*n*=3). *P<0.05 (one-way ANOVA). (**f**) Survival of WT MI mice at 28 days after injection, showing the ACM-A treatment can maintain a significantly higher animal survival than all the other treatments. The animal number (n) was 31, 29, 38, 31 and 24 for Saline, Single, Bare-A, ACM-A and ACM, respectively. *P<0.05 (one-way ANOVA). (**g**) Ejection fraction measured by PV loops showing the ACM-A treatment significantly improves the heart function after MI, compared with saline, Bare-A and ACM treatments. Error bars represent s.d. (*n*=4 for Single and *n*=3 for other groups). *P<0.05 (one-way ANOVA).

We further studied the degradability of the micromatrix *in vitro* by culturing the Bare-A *versus* ACM-A and observing their attachment in petri dishes. As shown in Supplementary Fig. 4a,b, cells detached from nearly all the Bare-A, spread out, and attached to the bottom surface of the petri dish after 1 day of culture. This occurred to 1.5, 35.4 and 71.7% of the ACM-A after 1, 2 and 3 days of culture, respectively (Supplementary Fig. 4a,c), as a result of the gradual degradation of the micromatrix. Furthermore, we conducted experiments to study the micromatrix degradation *in vivo* by labelling both oxidized (by default) and non-oxidized (NonOxi) alginate with indocyanine green (ICG, for non-invasive near infrared imaging, as shown in Supplementary Fig. 5a) to form the ACM-A-ICG and NonOxi-ACM-A-ICG, respectively. After intramyocardial injection into the peri-infarct zone of mice with MI (Fig. 4a), the ACM-A-ICG fluorescence was observed to gradually decrease and disappear over 3 days (Supplementary Fig. 5b). This indicates that the ACM degrades and diffuses away within 3 days after intramyocardial injection, which is slightly faster than its *in vitro* degradation due to the different microenvironment in the MI heart compared with the culture medium in petri dish. In contrast, the fluorescence of NonOxi-ACM-A-ICG stays over three days after intramyocardial injection because the non-oxidized (that is, regular) alginate degrades much slower than the oxidized alginate^36^. A similar trend was observed for the degradation rate when we subcutaneously injected the ACM-A-ICG and NonOxi-ACM-A-ICG into mice (Supplementary Fig. 5c). These *in vitro* and *in vivo* degradation data indicate that the ACM used in this study is biodegradable and provides a temporary micromatrix for the pre-differentiated cell aggregates.

It is worth noting that the aforementioned procedure for pre-differentiating the aggregated cells, releasing the pre-differentiated aggregates out of the microcapsules, and re-encapsulating them in ACM to form the ACM-A does not affect the high cell viability or the size and integrity of the aggregates (Fig. 1b; Supplementary Fig. 4b,c). The size (diameter) of aggregates was determined to be 128.9±17.4, 123.3±12.4 and 126.9±11.0 μm before releasing them from microcapsules, after releasing from microcapsules (for Bare-A) and after re-encapsulating them in ACM (for ACM-A), respectively.

### Injecting the re-encapsulated aggregates for therapy of MI

To investigate the safety and efficacy of the ACM-A as compared with Bare-A, bare single cells (Single), saline and materials alone (that is, ACM) for treating MI, we conducted intramyocardial injection of 2 × 10^5^ cells per animal in a total of 20 μl saline into the peri-infarct zone (injected at three different injection sites with equal volume, Fig. 4a) using a 28 gauge needle. This was done within 5 min after the anterior wall of the left ventricle turned pale following permanent ligation of the left anterior descending artery (LAD) at its proximal location (Supplementary Fig. 6a–d), to create a large-area MI (Supplementary Fig. 6e) in 8–12 week old male C57BL6/J mice. The mice are either wild-type (WT) with a normal immune system or caspase-recruitment domain 9 (*Card9*) knockout (KO) with deficient macrophage function^37^. This immediate injection of therapeutic cells is desired to minimize the damaging effect of MI. All surgical procedures were conducted under aseptic conditions. The treatments with Bare-A and single cells differentiate themselves from the others with the formation of a large mass of tissue that grows out of the heart (indicated by arrows in Fig. 4b). This kind of large grown-out tissue was observed in 60 and 57% of WT mice with the bare single cell (*n*=29) and Bare-A (*n*=38) treatment, respectively. In stark contrast, it was not detected in any of the WT mice treated with ACM-A (*n*=31), ACM (*n*=24) or saline (*n*=31) (Fig. 4c).

Further microscopic examination of the grown-out tissue revealed that it is a typical granuloma consisting of immune cells (macrophages and T cells) and fibroblasts within a loose tissue matrix (Fig. 4d, Supplementary Fig. 7a,b, and Supplementary Fig. 8, at 28 days post injection), indicating the outgrowth is a result of immune reactions. This kind of severe immune reactions might also lead to the clearance of transplanted cells by macrophages *in vivo*. This is because the retention/survival of implanted cells in the heart is significantly lower for the single cell (7.3±4.5%, consistent with that reported in the literature for single cell injection^5^) and Bare-A (17.6±6.3%) treatments than the ACM-A treatment (47.0±5.6%) at 28 days post injection (Fig. 4e and Supplementary Fig. 9). Furthermore, the Bare-A treated MI mice started to die at as early as 2 days post injection, while mortality was not observed in the ACM-A treatment group until 7 days after injection (Fig. 4f). Nearly 40% of the Bare-A treated MI mice died during the first 4 days, a known time frame for acute immune reaction to implanted cells^16^,^17^. Interestingly, while WT mice treated with single cells have a higher occurrence of granuloma, the overall size of granuloma is smaller than that of Bare-A treatment (Fig. 4b). This might be due to the reduced cell survival/retention and may explain the moderate animal mortality in the early time frame for the single cell treatment, compared with the Bare-A treatment.

Macrophages are known to regulate acute inflammation and remodelling in response to cardiac injury. To further understand this role of macrophages, we injected Bare-A into the MI hearts of *Card9* KO mice. Granuloma was observed in only ∼33% of the KO mice (Fig. 4c). These data suggest that macrophage-mediated acute immune reactions contribute to the occurrence of granuloma and possibly the early death of WT mice with MI treated with Bare-A.

The ACM-A treatment also significantly improves the cardiac function as indicated by ejection fraction measured by pressure–volume (PV) loops (Fig. 4g; Supplementary Fig. 10) and electrocardiography (Supplementary Fig. 11), compared with saline, Bare-A and ACM treatments. All groups with cells (Single, Bare-A and ACM-A) can partially restore the maximum and minimum rate of pressure change in the left ventricle (Supplementary Fig. 12a). The ACM-A treatment significantly improves the stroke volume and cardiac output compared with the saline and ACM treatments (Supplementary Fig. 12b), the time constant of isovolumic pressure relaxation (tau) compared with saline treatment (Supplementary Fig. 13a), and the left ventricle (LV) end-systolic and end-diastolic volumes compared with Bare-A and ACM treatments (Supplementary Figs 13b and 14). In addition, these functional parameters for the single cell and Bare-A treatments exhibit large variations, probably due to the occurrence of granuloma and strong immune responses in more than half of the mice with the two treatments (Fig. 4c).

### Cardiac regeneration *in situ* of the injected aggregates

**Figure 5:**
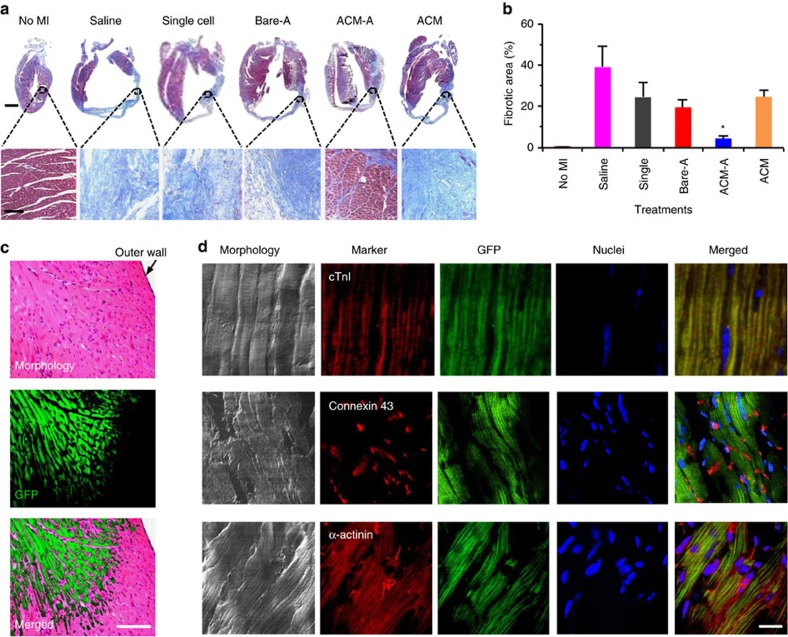
Cardiac regeneration *in situ* with the ACM-encapsulated pre-differentiated aggregates. (**a**) Low-magnification sagittal micrographs of Masson's trichrome stained tissue sections (top row) and zoom-in views of the left ventricular wall (bottom row) showing extensive fibrosis in the MI hearts treated with saline, materials alone (that is, ACM), single cells and Bare-A while it is minimal with the ACM-A treatment. Scale bar: 2 mm (top row) and 100 μm (bottom row). (**b**) Quantitative analysis showing the ACM-A treatment significantly reduces fibrosis in the MI heart. The *n*=3 and *P<0.05 (one-way ANOVA). (**c**) Micrographs of sectioned and H&E stained tissue (Morphology) in the MI zone of heart treated with the ACM-A showing green fluorescent CM-like cells (GFP) and their seamless integration with the neighbouring non-fluorescent native host tissue (Merged). Scale bar, 100 μm. (**d**) Immunohistochemically stained tissue from the MI zone of hearts treated with the ACM-A showing positive staining of CM (cTnI, connexin 43, and α-actinin, in red) markers co-localized with injected GFP cells. The striated pattern of the cardiac tissue with green fluorescence is evident. Scale bar, 10 μm. All tissues were harvested on day 28 after intramyocardial injection.

Furthermore, the ACM-A treatment significantly reduces fibrosis (Fig. 5a,b) compared with all other treatments at 28 days after injection. This should contribute to the high survival of the WT MI mice with the ACM-A treatment (Fig. 4f). In addition, all treated hearts are markedly larger than no-MI hearts, as a result of pathological hypertrophy in response to MI, trying to restore the stroke volume compromised by MI. However, the hearts from mice treated with ACM-A suffered less hypertrophy than mice with the other treatments, indicating that the ACM-A treatment facilitates the restoration of cardiac function of mice with MI. To find out if the significantly improved survival and the therapeutic benefit with the ACM-A treatment are partially a result of cardiac regeneration by the implanted cells *in situ*, we examined the heart tissue harvested from the MI mice implanted with ACM-A prepared using mESCs constitutively expressing green fluorescent protein (GFP) using our bioinspired approach. It was observed that extensive green fluorescence is evident in the MI zone (Fig. 5c) at 28 days post injection. Moreover, this green fluorescence co-localizes with the antibody staining of GFP expressed in live cells (Supplementary Fig. 15), suggesting that the implanted cells survived, detached from the aggregates and migrated into the MI zone. The implanted cells (expressing GFP) appear to be similar to CM with evident striations, suggesting further differentiation of the implanted cells (with early cardiac pre-differentiation) into more mature CMs under the *in situ* cardiac inductive chemical, mechanical and electrical cues in the heart after implantation. This is further supported by the expression of three typical markers of CMs including cardiac troponin I (cTnI), connexin 43 and α-actinin in the green fluorescent CM-like cells derived from the implanted cells (Fig. 5d). Moreover, the green fluorescent CMs integrated seamlessly with the neighbouring native CMs without green fluorescence (Fig. 5c,d), indicating the micromatrix was biodegraded allowing for timely integration of the implanted cell-derived CMs with the native cardiac tissue.

## Discussion

In this study, we report a bioinspired approach as illustrated in Fig. 1 to prepare PSCs for implantation. Microcapsules with a core–shell structure resemble the native physical configuration of pre-hatching embryos, the native home of totipotent-pluripotent stem cells. Probably because of the biomimetic nature, our previous studies show that the miniaturized and semi-closed culture in the core–shell microcapsules can better maintain the stemness of both pluripotent and multipotent stem cells compared with conventional culture in homogeneous liquid (medium) or hydrogel^29^,^35^,^38^,^39^. This is crucial to the expansion of stem cells with high quality and purity *in vitro* for the application of stem cell-based medicine, including but not limited to cardiac tissue regeneration for treating MI. However, the remaining steps of this bioinspired approach (including early cardiac pre-differentiation, dissolving the alginate hydrogel shell to release the pre-differentiated aggregates and re-encapsulation in ACM) mimic the phenomena of the processes of pre-differentiation, zona hatching and re-encapsulation in the trophoblast rather than the exact biological mechanisms of the processes that nature uses to prepare totipotent-pluripotent stem cells for implantation into the uterus wall. Biological processes of this natural procedure are tightly regulated and cell-induced and mediated. We do not intend to mechanistically mimic the processes in this work. However, our bioinspired approach is based on phenomenologically mimicking the natural procedure and does provide multiple advantages in preparing PSCs for implantation to treat MI and potentially other diseases as detailed below.

First, our bioinspired approach enables injectable delivery of cells with much improved efficiency. The contemporary practice of scaffold engineering is to make a scaffold first and then seed cells into the scaffold, which is extremely difficult (if not impossible) to utilize for making microscale constructs (that is, ACM-A) with densely packed cells as shown in Fig. 3. In stark contrast, our bioinspired approach resolves this challenge by making microscale cell aggregates first and then forming a micromatrix within and over the aggregates (Fig. 1b). An important advantage of this inverse (and bioinspired) scaffold engineering method is that it does not significantly change the construct size, which allows for intramyocardial injection of the constructs for minimally invasive delivery. Moreover, using the ACM-A densely packed with ∼1,500 cells in each aggregate for delivery is much more efficient than the contemporary method of sparsely encapsulating only 2–5 cells in ∼100 μm hydrogel microcapsules (without a core–shell structure) for injection into the heart^40^.

Second, our bioinspired approach minimizes immune responses to implanted cells. Severe immune responses were observed after implanting Bare-A and bare single cells as demonstrated by the formation of granuloma containing many macrophages and T cells (Fig. 4b–d; Supplementary Figs 7 and 8). CARD9 signalling plays an essential role in immune response due to its critical involvement in the function of macrophages, neutrophils and monocytes that are important for the acute phase of inflammation^37^,^41^,^42^,^43^. Therefore, the *Card9* KO mice with compromised macrophage function were used to determine whether macrophage-mediated events are the dominating mechanism of the immune responses observed. The occurrence of granuloma is reduced, but not completely eliminated in the *Card9* KO mice, indicating that T cells also play a significant role in the immune responses to the bare aggregates and single cells. With the ACM encapsulation, the occurrence of granuloma could be significantly reduced to 0, indicating the exceptional capability of immunoisolation by the ACM.

Interestingly, few T cells or macrophages could be observed in the heart treated with ACM-A at 4 weeks (Supplementary Fig. 8) even though the micromatrix (that is, ACM) was degraded within three days (Supplementary Figs 4 and 5). Due to the porous nature of the ACM, as shown in Fig. 3a, we propose that cells in the ACM-A after implantation could still communicate with the microenvironment in the host via soluble factors and gradually adapt to it. As a result, minimal immune responses would be evoked after the cells are exposed with the gradual degradation of the ACM. We therefore reason that the biodegradable ACM should temporarily isolate the encapsulated cells from contacting the host cells including macrophages, T cells, and possibly NK cells to achieve a temporary immunoisolation (or ‘local immunosuppression') effect. This may contribute to the significantly improved animal survival (∼80% after 28 days of injection), and elimination of granuloma in WT mice with the ACM-A treatment (Fig. 4c,f). Of note, animal survival for the saline control at day 28 after surgery was ∼48%, which is consistent with the literature for WT mice with a large-area MI^44^,^45^. This long-term impact of temporary immunosuppression/isolation was also recently reported elsewhere. For example, temporary systemic immunosuppression for up to 6 days was shown to mitigate immune rejection to bare pluripotent stem cells and their derivatives in the long term^11^,^12^,^13^. Our recent study shows that the impact of temporary immunoisolation for 3 days could last over 80 days in a T cell-based therapy of leukaemia^46^.

Probably due to the minimized immune response to the implanted cells in ACM-A, as well as the size of the aggregates (∼125 μm) preventing the cells from being carried away by blood perfusion, we observed significantly improved cell retention and survival with the ACM-A treatment at 4 weeks after implantation (Fig. 4c). Furthermore, we observed apparent engraftment of the implanted cells in the infarct region, as shown in Fig. 5c,d although the injection was done at the peri-infarct zone. These data suggest that cells pre-differentiated to the early cardiac stage may have migration potential, in contrast to mature cardiomyocytes with very limited migration potential. Furthermore, unlike mature cardiomyocytes that have a very specific role of beating in the heart, the pre-differentiated cells at the early cardiac lineage could potentially release factors to facilitate angiogenesis to promote their survival. In addition, the heart always beats, which might induce some (albeit insufficient alone) degree of fluid flow in the interstitial space of the cardiac tissue to improve the survival of the implanted cells migrating into the infarct zone. In addition, we used pre-differentiated mESCs in mouse MI model (that is, allogeneic model). This might also contribute to the improved engraftment when compared with xenograft models reported in the literature, where the human ESCs (hESCs) derived mature cardiomyocytes may have more difficulty integrating with the guinea pig or rat model since they are from different species^24^,^25^. It is worth noting that BMP-4 and bFGF have been used as the only two chemicals for specific cardiac (rather than the primitive mesoderm) differentiation of the pluripotent stem cells including the R1 mESCs used in this study^47^,^48^,^49^. In one of our previous studies, electrospray was also used to encapsulate the R1 mESCs in core–shell microcapsules for proliferation before cardiac differentiation^29^. These previous studies show that after the 3-day incubation in culture medium with BMP-4 and bFGF, it may take 4–11 more days for the pre-differentiated cells to mature further into beating cardiomyocytes when they are cultured in the base culture medium without BMP-4 and bFGF.

We did not observe any teratoma formation from these implanted cells retained and survived in the hearts of 98 WT mice treated with the pre-differentiated cells including ACM-A (31), Bare-A (38) and single cells (29) at 4 weeks after intramyocardial injection. Tissue slides from all the *in vivo* studies were carefully evaluated by an American Board of Pathology certified anatomic/clinical pathologist with extensive experience in evaluating both human and animal tissues without and with various diseases including cardiovascular diseases and cancer. It has been reported that teratoma can form in mouse models injected with pluripotent stem cells including the R1 mESCs used in this study in 3–4 weeks^8^,^22^,^23^,^24^,^25^. The aforementioned observation suggests that teratoma formation could be effectively eliminated by using our bioinspired method to prepare the mESCs for injectable delivery with early rather than mature cardiac pre-differentiation before implantation. However, potential teratoma formation needs to be monitored at a longer time points in future studies to confirm the observation.

In summary, our data show that ACM encapsulation of pre-differentiated microscale PSC aggregates significantly improves cell retention/survival after intramyocardial injection, and provides a temporary ‘local immunosuppression' for the cells to minimize cell injury and early animal death due to acute immune reactions. The implanted cells pre-differentiated to the early cardiac stage have a superb capability of regenerating cardiac tissue *in situ* via further direct differentiation guided by the cardiac-inducive chemical, mechanical and electrical cues in the heart into mature cardiomyocytes. This significantly reduces fibrosis and enhances cardiac function, which ultimately contributes to the significantly improved animal survival. This study indicates that the bioinspired engineering approach may be valuable to facilitate clinical applications of SCT for treating MI and possibly many other degenerative diseases.

## Methods

### Animals and materials

Male WT C57BL6/J mice (Jackson Laboratory, Bar Harbor, ME, USA) of 8–12 weeks, and age-matched *Card9* KO mice with C57BL6/J background^37^ (from Dr Xin Lin's laboratory at Department of Molecular and Cellular Oncology and Department of Immunology, University of Texas MD Anderson Cancer Center, Houston, TX, USA) were housed at constant temperature (22±2 °C) with a 12-h light/dark cycle. Mice were given standard lab chow and water *ad libitum*. All investigations in this study conform to the Guide for the Care and Use of Laboratory Animals published by the US National Institutes of Health and approved by the Institutional Animal Care and Use Committee at The Ohio State University.

Sodium alginate (plant cell culture tested and low viscosity, ∼240 kDa) was purchased from Sigma (St Louis, MO, USA) and purified by washing in chloroform and charcoal and dialyzing against deionized water, followed by freeze-drying^36^. The oxidization of alginate was performed by mixing sodium periodate (2 mM) with 1% (w/v) purified alginate for 24 h in the dark at room temperature, and then using an equivalent amount of ethylene glycol to stop the reaction, followed by 24 h of dialysis against deionized water with three water changes. ICG and FITC labelled alginate (with and without oxidization) was prepared by dissolving alginate or oxidized alginate (1.8% w/v) in 2-(N-morpholino) ethanesulfonic acid buffer (pH 4.7) and mixing with 9 mM 1-Ethyl-3-(3-dimethylaminopropyl) carbodiimide hydrochloride and 9 mM N-hydroxysulfosuccinimide (Sulfo-NHS). After stirring for 2 h at room temperature, 2 mM FITC (Sigma) or ICG amine (AAT Bioquest, Sunnyvale, CA, USA) was added to the solution and stirred for 18 h, followed by dialysis against distilled water for 24 h and freeze-drying to obtain the dry polymers of ICG or FITC labelled alginate (or oxidized alginate). Pharmaceutical grade chitosan of 80 kDa (∼95% deacetylation) was obtained from Weikang Biological Products Co. Ltd (Shanghai, China). The chitosan was dissolved in saline buffered with acetic acid at pH 6.7 and filtered through a 0.22 μm filter before use. The primary antibodies including ab47003 (to cTnI, rabbit polyclonal), ab18061 (to α-actinin, mouse monoclonal, clone 0.T.02), ab45932 (to cTnT, rabbit polyclonal) and ab11370 (to connexin 43/GJA1, rabbit polyclonal) were purchased from Abcam (Cambridge, MA, USA). The primary antibodies for NKX2.5 (sc376565, mouse monoclonal, clone A-3), CD3 (sc20047, for T cells, mouse monoclonal, clone PC3-188A), fibronectin (sc9068, rabbit polyclonal) and F4/80 (sc25830, for macrophages, rabbit polyclonal) were purchased from Santa Cruz Biotechnology (Santa Cruz, CA, USA). The primary antibody for GFP (cs2956, rabbit monoclonal, clone D5.1) was purchased from Cell Signaling (Danvers, MA, USA). All secondary antibodies were purchased from Life Technologies. All other materials were purchased from Sigma unless specifically mentioned otherwise.

### Cell culture and encapsulation in core–shell microcapsules

R1 mESCs with and without GFP were purchased from ATCC (Manassas, VA, USA). The GFP R1 cells (ATCC SCRC-1033) express GFP constitutively with the plasmid pEYFP from Clontech Laboratories, Inc. (Mountain View, CA, USA). The mESCs were cultured in feeder-free medium made of Knockout DMEM supplemented with 15% Knockout serum (Life Technologies), 1,000 U ml^−1^ leukaemia inhibitory factor, 4 mM l-glutamine, 0.1 M 2-mercaptoethanol, 10 μg ml^−1^ gentamicin, 100 U ml^−1^ penicillin and 100 μg ml^−1^ streptomycin in gelatin coated tissue culture flasks with daily medium change. To encapsulate the mESCs in microcapsules with a liquid core and hydrogel shell, they were detached from the flasks using 1 × trypsin/EDTA, washed by phosphate buffered saline (PBS), and then suspended at 5 × 10^6^ ml^−1^ in 0.25 M aqueous mannitol solution supplemented with 1% (w/v) sodium carboxymethyl cellulose as the core solution. The coaxial electrospray system including coaxial needle, pumps, voltage generator and collecting bath are shown in Supplementary Fig. 1. Before experiments, the coaxial needle, collection beaker and connection tubes were autoclaved to ensure sterility. All solutions used for electrospray were filtered using 0.22 μm filters. All the devices including pumps, syringes, cables of the voltage generator were wiped with 70% alcohol and then put in a biosafety cabinet/laminar flow hood designed for cell handling. The voltage generator was left outside the hood. The cell encapsulation experiments were performed inside the hood to avoid contamination. In brief, the core solution was pumped through the inner lumen (28G) of the coaxial needle at 47 μl min^−1^, while the shell solution consisting of 2% purified sodium alginate (w/v) in 0.25 M aqueous mannitol solution was pushed through the outer lumen (21G) at 60 μl min^−1^. Concentric drops generated by the core and shell flows were then broken up into microdroplets under a 1.8 kV electrostatic field and finally sprayed into the gelling solution made of 100 mM calcium chloride in deionized water. The distance between the needle tip and the top surface of CaCl_2_ solution in the gelling bath was 5.5 mm. The high viscosities of core and shell solutions, as well as the instant gelling kinetics of sodium alginate in the 100 mM CaCl_2_ solution ensure negligible mixture between the two aqueous solutions and therefore the formation of core–shell microcapsules. The resultant microcapsules were 315±31 μm in outer diameter^29^. After encapsulation, all the resultant cell-laden microcapsules were washed with 0.5 M mannitol solution and cultured in mESC medium with daily medium change. All cells were cultured at 37 °C in a humidified 5% CO_2_ incubator.

### Pre-differentiation of mESC aggregates

After 7-day culture in mESC medium, one integrated mESC aggregate was formed in the core of each microcapsule. For pre-differentiation of the mESC aggregates towards mesoderm and further the early cardiac lineage, cell aggregates were cultured in cardiac induction medium with regular DMEM supplemented with 25 ng ml^−1^ BMP-4, 5 ng ml^−1^ bFGF, 100 U ml^−1^ penicillin and 100 mg l^−1^ streptomycin for 3 days in the core–shell microcapsules to prevent attachment on the culture plate. To characterize gene expression after cardiac induction, aggregates were released from the microcapsules by dissolving the alginate hydrogel shell using 55 mM sodium citrate for 30 s and then washed by PBS for 3 min. The pre-differentiated aggregates together with undifferentiated ones (as control) were then homogenized and used for RNA isolation following the manufacture's instruction with the RNeasy Plus Mini Kit (Qiagen). The quality of the extracted RNA was then examined by its A_*260*_/A_*280*_, A_*260*_/_*230*_ and 28S/18S ribosomal RNA (rRNA) ratio. The microarray was conducted using the Clariom D assays for mouse (Affymetrix, Santa Clara, CA, USA) on GeneChip Hybridization Oven 645 and analysed using the Affymetrix Transcriptome Analysis Console (TAC) Software by technicians in The Genomics Shared Resource in The Ohio State University Comprehensive Cancer Center. A complete list of all genes investigated with the Clariom D assays can be found at: http://www.affymetrix.com/estore/catalog/prod870009/AFFY/Clariom+D+assays,+mouse#1_3. For qRT-PCR studies, the synthesis of complementary DNAs (cDNAs) was conducted using the iScriptTM cDNA synthesis kit (Bio-Rad, Hercules, CA, USA) and quantitative PCR analysis was performed using a Bio-Rad CFX96 real time PCR machine. In brief, relative gene expression was calculated using the ΔΔCt method built in the Bio-Rad software and the expression of the early cardiac gene *Nkx2.5* (F: 5′-GATGGGAAAGCTCCCACTATG-3′ and R: 5′-GAGACACCAGGCTACGT CAATA-3′) was studied.

For immunostaining of NKX2.5 protein, the mESC aggregates before (as control) and after pre-differentiation were either dissociated using 1 × trypsin/EDTA into single cells or kept as intact aggregates for fixation using 4% paraformaldehyde for 1 h at room temperature. The fixed single cells or aggregates were incubated first with 3% bovine serum albumin (BSA, to block non-specific binding) in PBS with 0.1% TritonX-100 (for permeabilization of the membrane within cells) for 30 min at room temperature, and then with NKX2.5 primary antibody (1:200 dilution) overnight at 4 °C, washed three times using PBS, incubated with secondary antibody (1:200 dilution) for 1 h at room temperature. The samples were further washed three times using PBS before microscopic examination using an Olympus FV1000 confocal microscope and flow cytometry analysis using a BD (Franklin Lakes, NJ, USA) LSR-II Flow Cytometer and FlowJo software. This protocol was also used for other antibodies including OCT-4 (1:200 primary antibody dilution), NANOG (1:200 primary antibody dilution) and cTnT (1:100 primary antibody dilution). This is the protocol suggested by the manufacturers (Abcam and Santa Cruz) for the primary and secondary antibodies used for immunohistochemical staining and flow cytometry.

### Re-encapsulation of the pre-differentiated aggregates

After aggregates formed after 7-day culture, we collected the aggregate-laden microcapsules from each culture dish by pipetting with a 15 ml serological pipette into a 15 ml centrifuge tube. Due to gravity, the aggregate-laden microcapsules could sink down at the bottom of the collection tube in 5 min. The supernatant was then removed using 5 ml serological pipettes, and the aggregate-laden microcapsules were rinsed with 5 ml of PBS. The latter was done by gently tilting the tube for mixing and by allowing the microcapsules to sink down at the bottom of the tube as a result of gravity before removing the supernatant by pipetting. A total of 1 ml of 55 mM isotonic sodium citrate was then added into the tube to dissolve the alginate hydrogel shell of the microcapsules in 30 s, followed by removing the supernatant containing sodium citrate and dissolved alginate by pipetting. The aggregates were then washed using 5 ml of PBS in the same way as that aforementioned for washing the aggregate-laden microcapsules. Unlike trypsin or collagenase, the process of dissolving alginate hydrogel using sodium citrate is quick and gentle and it does not affect the integrity of aggregates. For ACM encapsulation of the resultant cell aggregates, alginate and chitosan solutions were dissolved in saline (0.9%) with a pH at 7.2 and 6.7 (this is necessary to dissolve chitosan), respectively. The size (diameter) of aggregates was determined by measuring the average diameter at three different locations on each aggregate with a 120-degree interval. Chitosan is a positively charged polymer that can be attracted to the cells in the aggregate because their plasma membrane is negatively charged. The same electrostatic interaction applies to the complexation of alginate with chitosan and vice versa (alginate is negatively charged). Alginate and chitosan are both naturally derived polymers with high biocompatibility and can gradually degrade into nontoxic oligosaccharide and glucosamine (amino sugar), respectively^46^,^50^,^51^,^52^. The materials alone (ACM) control was achieved by processing alginate microbeads of similar size to the cell aggregates in the same way as that for forming the ACM within the cell aggregates. The ACM encapsulated aggregates were then used for either *in vitro* studies to determine the existence and degradation of ACM or *in vivo* transplantation into mice. In brief, the aggregates encapsulated in ACM of oxidized or regular alginate were plated on gelatin-coated dishes and maintained in regular DMEM with 20% FBS. The ratio of attached to total aggregates were calculated to determine the percentage of aggregate attachment. Non-encapsulated aggregates were studied in the same way to serve as control.

For morphological characterization of the bare *versus* ACM encapsulated pre-differentiated aggregates using scanning electron microscopy (SEM), the aggregates were fixed using 2.5% glutaraldehyde in phosphate buffer overnight. After the standard procedures of dehydration in ethanol and drying using hexamethyldisilazane, the samples were sputter coated using a Cressington 108 sputter coater at 17 mA for 120 s before examination using an FEI Nova NanoSEM 400 scanning electron microscope. For visualization of ACM, the mESC aggregates were encapsulated in ACM with FITC-labelled alginate using the aforementioned procedure, and the encapsulated aggregates were further stained for nuclei using 5 μM Hoechst 33342 for 15 min before examination using an Olympus FV1000 confocal microscope. In addition, ∼150 μm microbeads made of 2% (w/v) oxidized alginate were generated by electrospray and then soaked in chitosan and alginate solutions in the same way as preparing the ACM encapsulated pre-differentiated aggregates to serve as the materials alone control. For all *in vitro* studies, cells were cultured at 37 °C in a humidified 5% CO_2_ incubator.

To quantify the elastic modulus of mESC aggregates, nanoindentation was performed using an integrated system consisting of an Olympus IX81 inverted optical microscope and an Asylum MFP-3D Bio atomic force microscope (AFM). To reduce any potential transient thermal effects on cantilever deflection, the cantilever was kept at the same temperature as the mESC aggregates before obtaining force curves. After calibrating the cantilever sensitivity, the thermal tuning method was used to determine the cantilever's spring constant. Multiple mESC aggregates were then suspended in PBS and allowed to settle onto the glass bottom of petri dishes (Fluorodish, World Precision Instruments, Inc.). Individual aggregates were identified and positioned underneath the cantilever tip using the inverted optical microscope. Force *versus* indentation curves were then generated using the deflection data from the contact point between the sample surface and the cantilever tip. Colloidal probes (2 μm diameter polystyrene beads) were chosen for their low spring constant (0.32 N m^−1^) and spherical tip geometry, which are commonly used for indenting soft biological samples. Force curves at a distance of 4 μm were acquired at 0.5 Hz to reduce the viscoelastic effects that can occur when using larger approach velocities. A relative trigger force of no >10 nN was also used to ensure that indentations did not produce any unwanted effects from the underlying substrate, as determined by preliminary testing over a wide range of indentation depths. The resulting curves were then fit using a Hertz model built into the AFM software. Multiple force curves (at least 10) were taken for each sample in order to ensure that a consistent elastic modulus was produced. The resulting data sets were then combined and evaluated using statistical analysis for comparison.

### Surgical procedure and intramyocardial injection

To perform permanent ligation of LAD, mice were initially anesthetized by 3% isoflurane inhalation, intubated with a 20G intravenous catheter and ventilated with a mixture of O_2_ (0.3 l min^−1^) and 1.5–2% isoflurane (tidal volume of 250 μl and 120 breaths min^−1^) with a mouse respirator (Harvard Apparatus, Holliston, MA, USA). Animals were placed in a right lateral decubitus position and a left thoracotomy was then performed through the left fourth intercostal space by cutting pectoralis muscles transversely to expose the thoracic cage. After removal of the pericardium, the left anterior descending artery was visualized and a 6–0 silk ligature was placed ∼1 mm from origin of the vessel. The ligature was confirmed to be successful when the anterior wall of the left ventricle turned pale.

Triphenyl tetrazolium chloride staining was conducted at 24 h after surgery to confirm the ligation. In brief, ∼200 μl of 10% Phthalo Blue were slowly injected into the aorta to stain the heart. The heart were then rapidly collected and washed in 30 mM KCl to cease the heart beating and allow for more consistent sectioning. The heart was then frozen down for at least 4 h at −20 °C before cutting them into slices of 1 mm. The heart tissue slices were incubated with 2% triphenyl tetrazolium chloride at 37 °C for 40 min. Further fixation of the stained slices with 10% formaldehyde overnight was performed, to increase the contrast between the infarct area and the normal tissue.

Injection of cell aggregates and other control groups was performed as illustrated in Fig. 4a. The single cells were obtained by dissociating Bare-A with 1 × trypsin/EDTA, similarly to detaching 2D cultured cells. The number of cells in each aggregate was determined by dissociating 50 aggregates using trypsin and counting the dissociated cells manually. For tracking cell differentiation *in vivo*, the same cells with GFP were used. Mice randomly received a total of 20 μl saline or saline containing single cells, Bare-A, ACM-A or ACM via three injections given at three different sites in the periphery of infarcted tissue using a 28G needle within 5 min after the anterior wall of the left ventricle turned pale. The size (diameter) of the aggregates in this study was measured to be 123.3±12.4 μm before ACM encapsulation and 126.9±11.0 μm after ACM-encapsulation. Therefore, adding 3 times s.d. (or 3 sigma) to the average equals ∼160 μm, which is still much smaller than the inner diameter (184 μm) of a 28G syringe needle. Assuming normal distribution of the size, there is only 0.15% possibility for the cell aggregates to be bigger than 160 μm by the three-sigma rule. In addition, the cell aggregates are soft and deformable, which may help to push them through the syringe needle. Indeed, we did not notice any difficulty during injection of the cell aggregates during our experiments. In addition, injection volumes of 20–30 μl were used in previous studies with mouse models^8^,^23^, and our animal survival is not significantly different from that reported in the literature for the treatment with saline^53^,^54^,^55^. Moreover, with saline, ACM-A and ACM treatments in 20 μl of injection volume, we did not observe any granuloma formation. Therefore, the effect of a total of 20 μl of injection volume on granuloma formation and animal survival is insignificant in this study. After injection, the chest wall and skin were closed. The level of anaesthesia (end-tidal concentration of isoflurane: 1–2%) was adjusted according to the surgical stimulation, by monitoring signs of movement. As soon as the anaesthesia was stopped, the animals woke up quickly (normally in ∼1–2 min). Sham operations were performed on 10 mice without LAD occlusion. On each day of conducting surgery, 4–6 mice were randomly assigned to the six groups including saline, single cells (Single), Bare-A, ACM-A and ACM. We plotted the data of heart rate in Supplementary Fig. 16, which shows no significant difference among different groups before *versus* after surgery. In total, 29 or more mice received treatment with saline, single cell, Bare-A or ACM-A, and 24 mice were treated with ACM (materials alone). The sample size was chosen to ensure adequate power to detect the difference between the saline control and ACM-A groups^56^. In brief, we estimated that the saline group had a survival rate of ∼35% from pilot studies and the literature^53^,^54^,^55^, the hypothetical survival rate of ACM-A from pilot studies was 80%, the probability of type I error (α)=0.05, the power (1−*β*)=0.8, and ratio of sample sizes is 1, which gives sample size required for each group to be at least 24. Mice were euthanized by CO_2_ exposure at 28 days, and heart samples were harvested for further analyses. No blind method is involved for the animal study. The total number of mice used in different studies is summarized in Supplementary Table 1.

The *in vivo* degradation of ACM were studied by encapsulating cell aggregates with ACM labelled by ICG and then injecting them into hearts of animals with the same surgical procedure as aforementioned. Bare-A and NonOxi-ACM-A-ICG were also injected as negative and positive controls, respectively. Hearts were collected at days 0 (1 h), 1 and 3 and the ICG fluorescence was monitored using a PerkinElmer (Waltham, MA, USA) IVIS imaging instrument with excitation at 780 nm and an 831 nm filter. Subcutaneous injection was also performed with injection of Bare-A, NonOxi-ACM-A-ICG and ACM-A-ICG, and the ICG fluorescence was observed noninvasively on days 0 (1 h), 1 and 3.

### Characterization of cardiac function *in vivo*

Post-surgery mice were anesthetized with 2% isoflurane in 100% oxygen. The chest area was shaved and ultrasound coupling gel was liberally applied to the left chest wall. Two-dimensional and M-mode echocardiographic images were recorded and analysed by a Vevo 2100 High-Resolution *in vivo* Imaging System with a MS400 transducer (VisualSonics, Toronto, ON, Canada). Images were obtained in a parasternal short and long axis view. At the same time, the limbs of mice were attached with three electrical leads (lead I: right front foot, lead II: left rear foot and lead III: left front foot) connected to the Vevo 2100 High-Resolution *in vivo* Imaging System for recording the electrocardiogram and heartbeat during the whole echocardiography examination process.

Since the data from echocardiography could be subject to variations due to experimental conditions, we further examined cardiac function by hemodynamic studies using PV loop measurements as the gold standard for quantifying cardiac function^57^,^58^. A mouse PV loop analysis system was used for the assessment of cardiac function. Briefly, 28 days after left coronary ligation, all mice with no MI and treated with saline, single cells, Bare-A, ACM-A and ACM were anesthetized with 2% isoflurane and oxygen at a flow rate of 0.4 l min^−1^. A Millar (Houston, TX, USA) tip conductance catheter (Model SPR-893, 1.4 Fr.) was inserted into the right carotid artery, and further advanced into the left ventricle (LV). Baseline zero reference was obtained by placing the sensor in isotonic saline. After recording the basal hemodynamic parameters, a series of PV loops were generated using an ADInstruments (Colorado Springs, CO, USA) Power-Lab system connected to the Millar catheter. All the measurement and characterization were determined from the PV loop data using ChartPro Software (AD Instruments).

### Histomorphological and immunostaining analyses

Gross examination of the distribution of cells with GFP in MI hearts treated with saline, single cells, Bare-A, ACM-A and ACM was done by Zeiss MosaiX tiling and stitching bright-field and green fluorescence images taken using the same exposure time. To estimate the retention and survival of injected cells, a method similar to that reported elsewhere^8^,^59^,^60^ was used with slight modification. Briefly, hearts injected with GFP cells (Single, Bare-A and ACM-A) and treatments without cells (saline and ACM) were harvested at 4 weeks after the injection and then embedded in OCT (Sakura Finetek, Torrance, CA, USA) to freeze at −80 °C for 1 h before cryo-sectioning. The cryo-sectioning was conducted as follows: 10 locations were chosen from the apex to the ligation site with an even interval, and then 2–3 sections of 10 μm in thickness (*T*) were cut at each location. All the resultant 20–30 sections were further stained with DAPI for visualizing nuclei, and then examined under a Zeiss (Oberkochen, Germany) Axio Observer.Z1 microscope with fluorescence capability. GFP positive cells (*N*) were counted on each section and averaged among the 20–30 sections. The length of the infarct zone (*L*) was measured using a ruler. The retention/survival rate (RR) was calculated as follows:


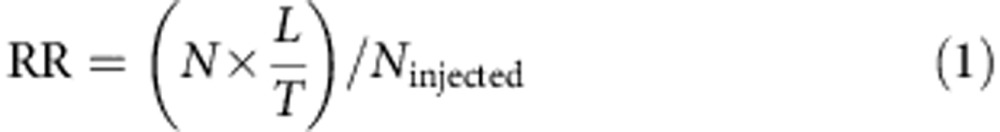


where *N*_injected_ is 0.2 × 10^6^. From Fig. 5a, it can be seen that the infarct zone is from the apex to the site of ligation. Therefore, the retention rate calculation was based on this area. We also checked the left ventricular wall of mice with no MI and without any treatment in the same way, and did not observe any green fluorescence.

For histomorphological analysis, hearts were harvested on day 28 post treatments, fixed in 10% neutral buffered formalin for 24 h at 4 °C, and embedded in paraffin. Both transverse and sagittal sections of 5 μm thick were then cut using a microtome. For H&E staining, sections were stained in hematoxylin 2 for 8 min and then washed by water, dipped in 1% acid alcohol and 1% ammonium hydroxide, stained in Eosin Y for 1 min, dehydrated with graded alcohols and mounted on slides. For Masson's Trichrome staining, sample sections were fixed using Bouin's Fixative overnight at room temperature and then stained in Weigert's Iron hematoxylin for 10 min. After rinsing with water for 10 min, the sections were dipped in Biebrich Scarlet Acid Fuchsin for 2 min and then in 5% Phosphomolybdic/Phosphotungsic acid for 15 min, followed by dipping for 5 min in 2.5% Aniline blue and 1% Glacial Acetic Acid, respectively. The sections were further dehydrated with graded alcohols and mounted on slides. For slides stained with Masson's trichrome, ImagePro 6.2 software was used to calculate the percentage of area that has increased collagen content indicating fibrosis of the cardiac tissue.

Immunofluorescence staining of macrophages and T cells in granulomas of single cell and bare-A treated mouse hearts was performed. In brief, the granuloma tissues were taken from the hearts and then cryo-sectioned into 5 μm of sections using a Leica CM1510S Cryostat and fixed with 4% paraformaldehyde. Sections were immunostained against specific markers including F4/80 (for macrophages, 1:100 dilution), CD3 (for T cells, 1:50 dilution), fibronectin (for fibroblasts, 1:200 dilution), as well as their corresponding secondary antibodies (1:200 dilution). Before final examination, cell nuclei were stained with 5 μM Hoechst for 15 min at room temperature. The images were captured with an Olympus FV1000 confocal microscope.

To determine the capacity of *in situ* differentiation for cardiac regeneration of the cells in ACM-A as well as their capability of integration with the host tissue, heart samples collected at 28 days post intramyocardial injection were cryo-sectioned into 10 μm of sections using the Leica CM1510S Cryostat and fixed with 4% paraformaldehyde. Sections were immunostained against cardiac specific markers including cTnI (1:200 dilution), α-actinin (1:200 dilution), connexin 43 (1:200 dilution), as well as their corresponding secondary antibodies (1:200 dilution), according to the manufacturer's instructions. Before final examination, cell nuclei were also stained with 5 μM Hoechst for 15 min at room temperature. The images were captured using an Olympus FV1000 confocal microscope.

For GFP staining, the fixed and paraffin-embedded heart tissues were cut into 5 μm of sections. All sections were deparaffinized and hydrated in decreasing concentrations of ethanol. Antigen retrieval reagent (HK080-9K, BioGenex, Fremont, CA, USA) was used according to the manufacturer's instructions. The heart sections were incubated with rabbit anti-GFP primary antibody (1:200 dilution) for 3 h at room temperature in blocking buffer (2% BSA in PBS). Following washing with PBS, tissue sections were further incubated with goat anti-rabbit (H+L) Alexa 568 secondary antibody (Life Technologies, A-11011, 1:500 dilution) for 1 h at room temperature. The cell nuclei were visualized by staining the tissue with Vectashield Mounting Medium with DAPI (H-1500, Vector Laboratories, Burlingame, CA, USA). Images for GFP staining were taken using an Olympus FluoView FV1000 confocal Microscope.

### Statistical analysis

Student's two-tailed *t*-test assuming equal variance was performed using Microsoft Excel to determine the statistical significance for *in vitro* studies assuming normal distribution and equal variances, including flow cytometry, AFM nanoindentation and qRT-PCR. Each experiment was independently repeated at least three times. Data are presented as mean±s.d. unless specifically indicated otherwise. The fold change of genes in microarray study was analysed using the Affymetrix Transcriptome Analysis Console (TAC) Software. The survival curve was plotted and analysed using Prism (v6.0, GraphPad software, San Diego, CA). To determining the statistical significance of granuloma formation among the different groups, a chi-square test was performed using Prism. For the analysis of *in vivo* results, unpaired *t*-test or ANOVA was conducted using Prism. The data analysed by ANOVA follows normal distribution and each sample was independent and random with similar variances between different groups. In all cases, a *P* value less than 0.05 was considered to be statistically significant.

### Data availability

The microarray data are deposited to Gene Expression Omnibus (GEO: http://www.ncbi.nlm.nih.gov/geo/) and publically available with accession code: GSE87066. All the other data supporting the findings of this study are available from the corresponding authors on request.

## Additional information

**How to cite this article:** Zhao, S. *et al*. Bioengineering of injectable encapsulated aggregates of pluripotent stem cells for therapy of myocardial infarction. *Nat. Commun.* **7,** 13306 doi: 10.1038/ncomms13306 (2016).

**Publisher's note:** Springer Nature remains neutral with regard to jurisdictional claims in published maps and institutional affiliations.

## Supplementary Material

Supplementary Information Supplementary Figures 1-16 and Supplementary Table 1.

Supplementary Movie 1 Micromatrix of alginate and chitosan formed within cell aggregate. The micromatrix is visualized by using alginate labeled with green fluorescence to form the micromatrix and imaging with confocal fluorescence microscopy.
