# Supplementary material for: Bioengineering of injectable encapsulated aggregates of pluripotent stem cells for therapy of myocardial infarction
Source: Nat Commun. 2016 Oct 27;7:13306. doi: 10.1038/ncomms13306 (PMC5095349; doi:10.1038/ncomms13306)
Supplement: Supplementary Information — Supplementary Figures 1-16 and Supplementary Table 1. [file ncomms13306-s1.pdf]

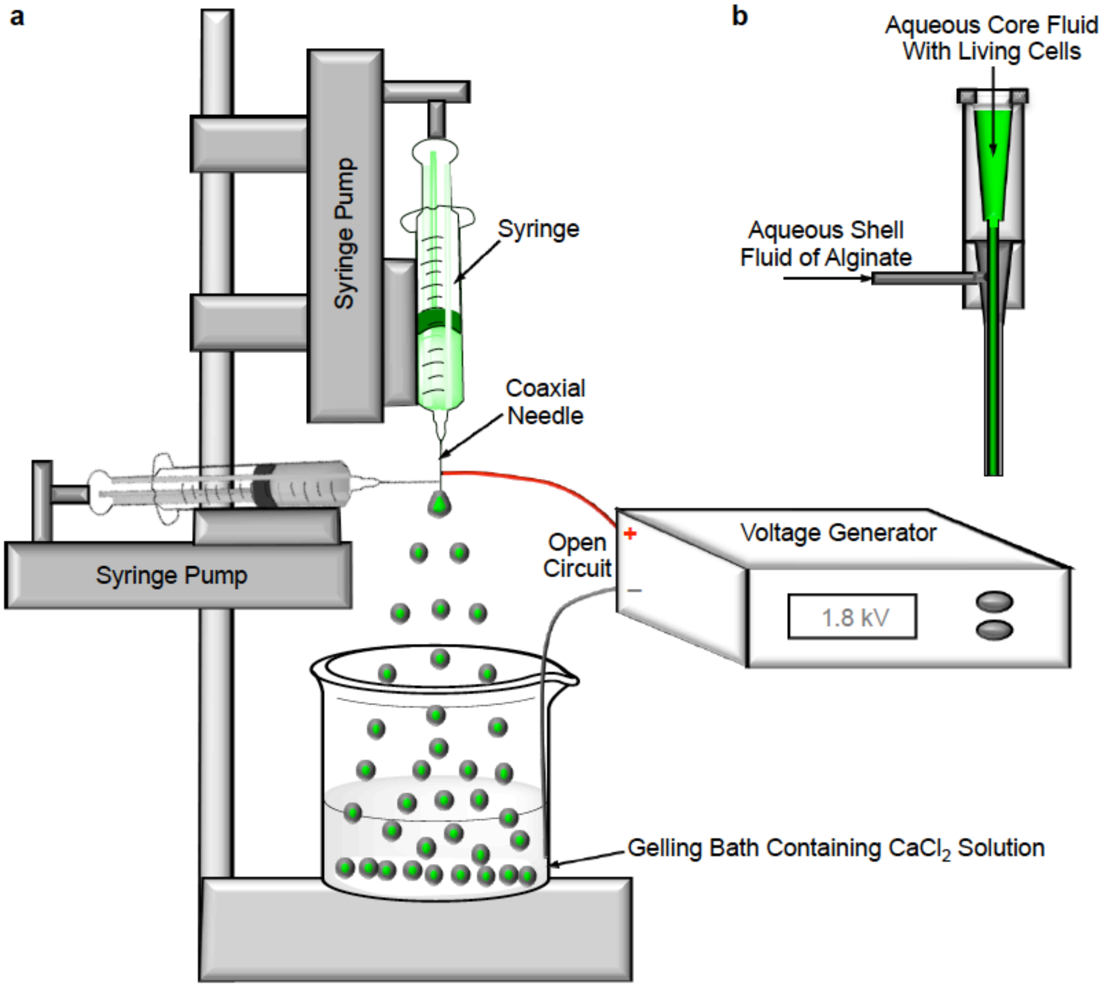

Supplementary Figure 1. A schematic illustration of the setup for coaxial electrospray. a, A schematic overview of the setup for coaxial electrospray to encapsulate murine embryonic stem cells (mESCs) in core-shell microcapsules. **b**, A zoom-in cross-sectional view of the coaxial needle. The core solution (green) with live mESCs and the shell solution of alginate (gray) are pumped through the inner and outer lumen in the coaxial needle, respectively, resulting in the formation of concentric drops at the needle tip. The drops are then broken up into microscale droplets under an open electrostatic field (that is, with no electric current), and sprayed into the gelling bath that contains divalent calcium cations for instant gelling of alginate to form the hydrogel shell of the resultant microcapsules.

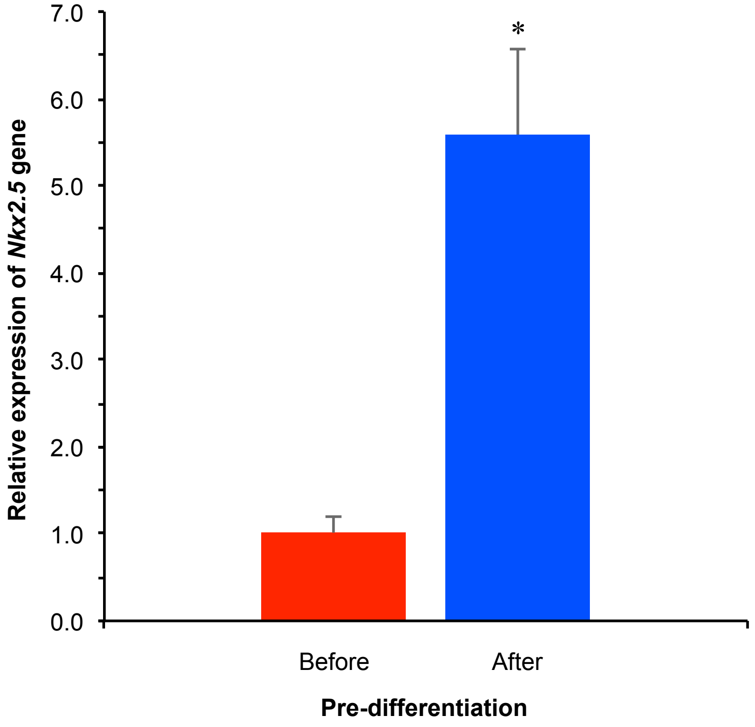

Supplementary Figure 2. Expression of the early cardiac marker gene *Nkx2.5*. The gene expression was quantified by quantitative reverse transcription polymerase chain reaction (qRT-PCR). The data show significantly up-regulated expression of the early cardiac marker gene in the aggregated cells after pre-differentiation. Error bars represent standard deviation (s.d., n=3). *, $p < 0.05$ (Student's two-tailed t test)

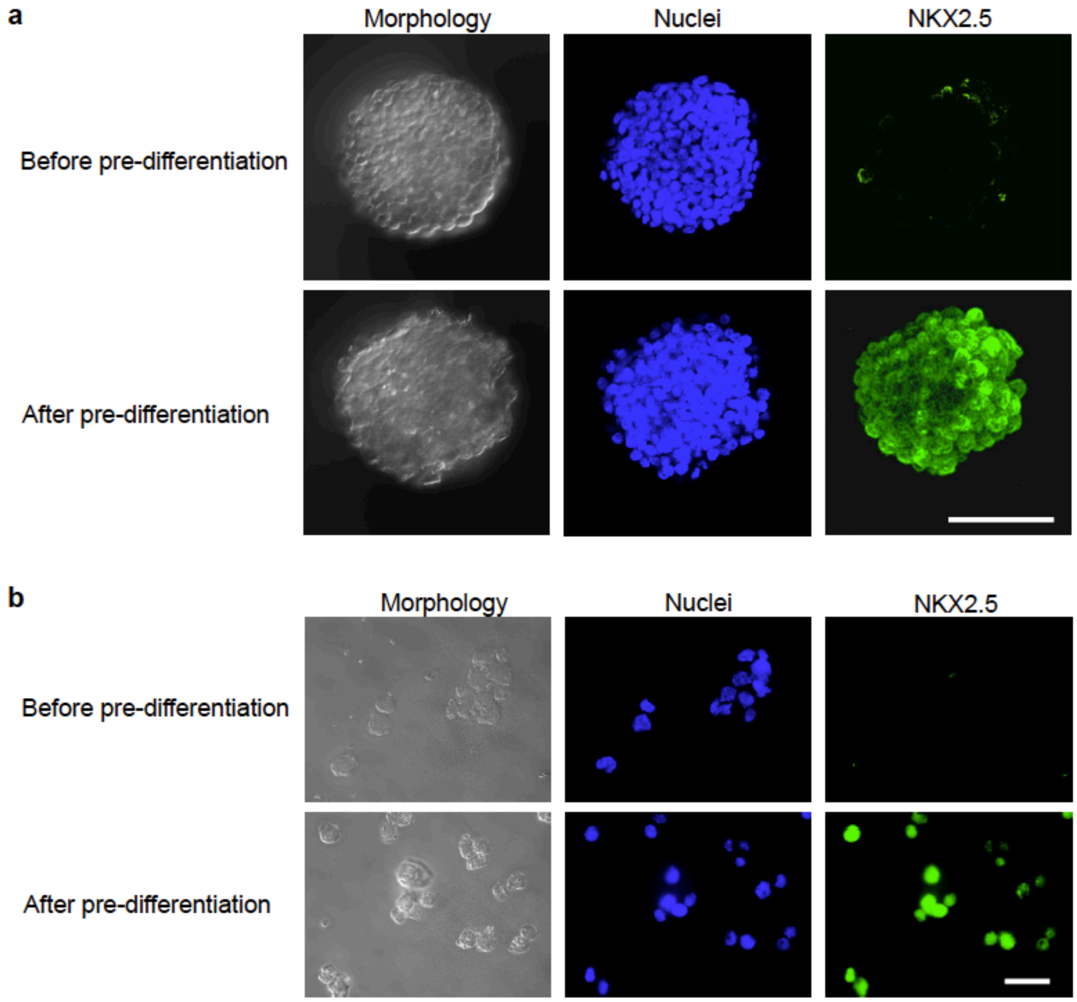

Supplementary Figure 3. Immunohistochemical staining of the early cardiac protein marker NKX2.5. a, Staining done over intact aggregates before and after pre-differentiation. Scale bar: 100 μm. **b**, Staining done on single cells disassociated from their aggregates before and after the pre-differentiation. Scale bar: 20 μm. The data show successful pre-differentiation of the aggregated mESCs into the early cardiac stage. All the fluorescence images shown are confocal, which means the views are cross-sections through the aggregates or single cells. Although the NKX2.5 protein functions in the nuclei, we also observed its stain in the cytoplasm, probably because the protein is synthesized in the cytosol. The striated pattern is difficult to discern in the pre-differentiated cells because the cells were pre-differentiated into the early cardiac stage rather than mature cardiomyocytes.

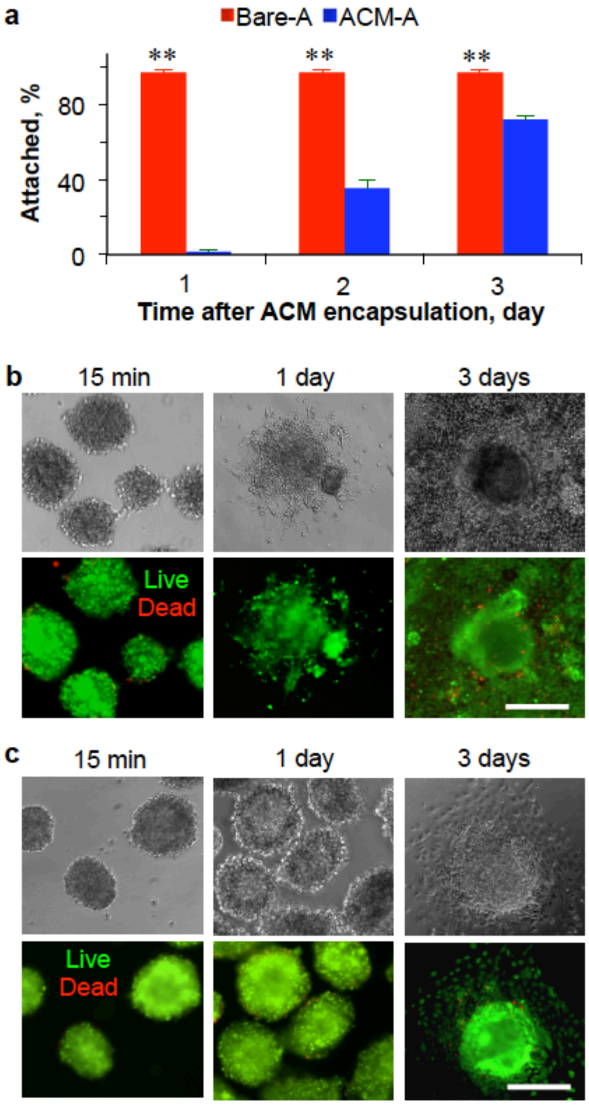

Supplementary Figure 4. Biodegradation of the micromatrix of alginate and chitosan *in vitro*. a, Quantitative data showing alginate-chitosan micromatrix (ACM) encapsulation slows down the attachment of the pre-differentiated cells from their aggregates when cultured in tissue culture petri dishes. Error bars represent s.d. (n=3). **, $p < 0.01$ (Student's two-tailed t test). Typical differential interference contrast (DIC) and fluorescence images showing the morphology and high cell viability of bare (**b**) and ACM-encapsulated (**c**) pre-differentiated aggregates cultured in petri dishes for 15 min, 1 day, and 3 days. Scale bar: 100 µm. Cells gradually detached from ACM-encapsulated aggregates and attached on petri dish as a result of the gradual degradation of the oxidized alginate in the ACM. Bare-A: Bare pre-differentiated aggregates. ACM-A: ACM-encapsulated, pre-differentiated aggregates.

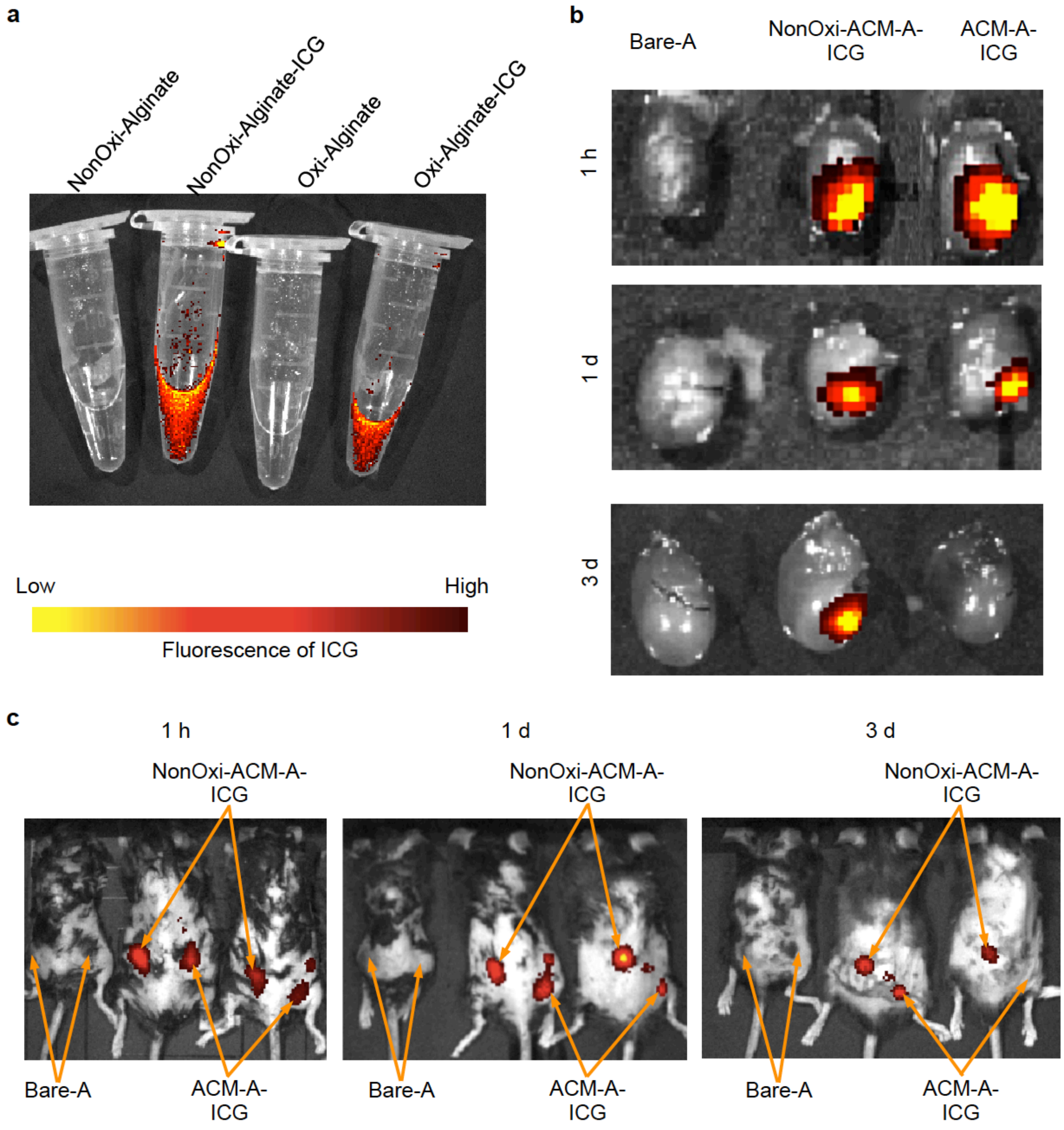

Supplementary Figure 5. Biodegradation of the micromatrix of alginate and chitosan *in vivo*. a, IVIS images showing successful labeling non-oxidized (NonOxi) alginate and oxidized (Oxi) alginate (by default and used for making the alginate-chitosan micromatrix or ACM in this study) with indocyanine green (ICG). **b,** ICG fluorescence in the MI heart of mice injected with Bare-A, NonOxi-ACM-A-ICG made of ICG labeled non-oxidized alginate, and ACM-A-ICG made of ICG labeled oxidized alginate. No fluorescence was observed in the hearts treated with Bare-A on days 0 (I h), 1, and 3. The fluorescence in the hearts treated with ACM-A-ICG reduced over time and disappeared on day 3, indicating the ACM gradually degraded over three days *in vivo* in the heart. The degradation of NonOxi-ACM-A-ICG is slower than that of ACM-A-ICG and the fluorescence was still observable on day 3 in the heart. **c,** ICG fluorescence in mice subcutaneously injected with Bare-A, NonOxi-ACM-A-ICG, and ACM-A-ICG. The trend of degradation for the subcutaneously injected NonOxi-ACM-A-ICG and ACM-A-ICG is similar to the intramyocardially injected NonOxi-ACM-A-ICG and ACM-A-ICG. The former degrades slower than the latter.

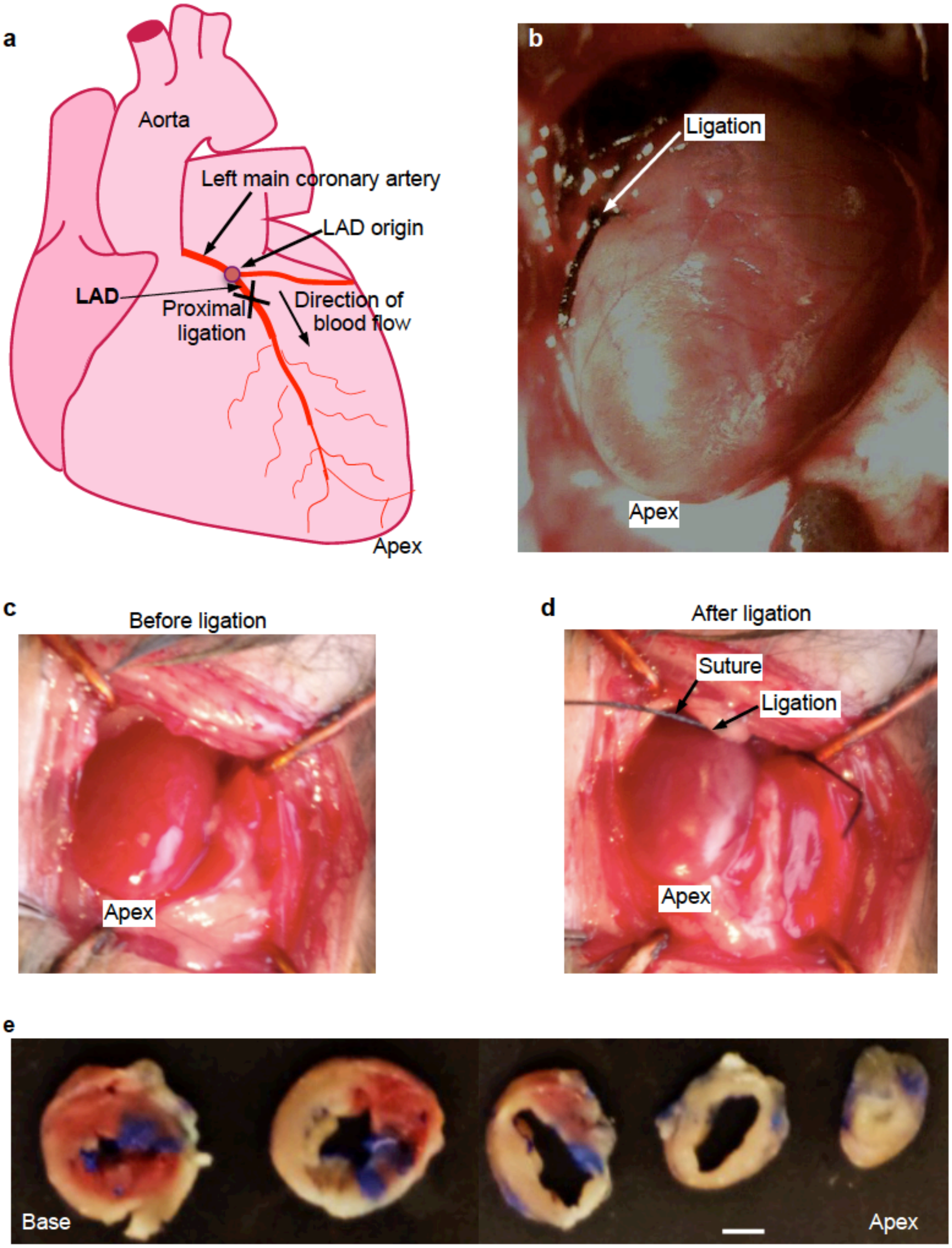

Supplementary Figure 6. The surgical procedure of permanent ligation to simulate myocardial infarction. a, A schematic illustration of permanent ligation of the left anterior descending artery (LAD) at its proximal location. Ligation at the proximal location results in large-area myocardial infarction (MI). **b**, A picture of the mouse heart collected after surgery, showing the location of ligation as compared to the sketch in panel a. **c,** A picture of the heart *in situ* before ligation showing fresh red on the outer surface of the anterior wall of the left ventricle. **d**, A picture of the heart *in situ* after ligation of the LAD at the proximal location, showing the pale appearance of the anterior wall of the left ventricle due to the lack of blood supply indicating successful ligation. Of note, the long suture is to be cut before closing the chest. **e**, Representative photographs of the cross-sectional sections of a heart from base to apex stained with tetrazolium chloride and blue at 24 h after ligation, showing non-ischemic/normal regions (blue), area at risk (red), and area with infarction (white). The infarct (the total white area in all the cross-sections) was quantified to be 65.0 ± 1.6% (mean ± s.d., n=3) of the total area of all the cross-sections, which is considered as large-area infarct. Scale bar: 3 mm

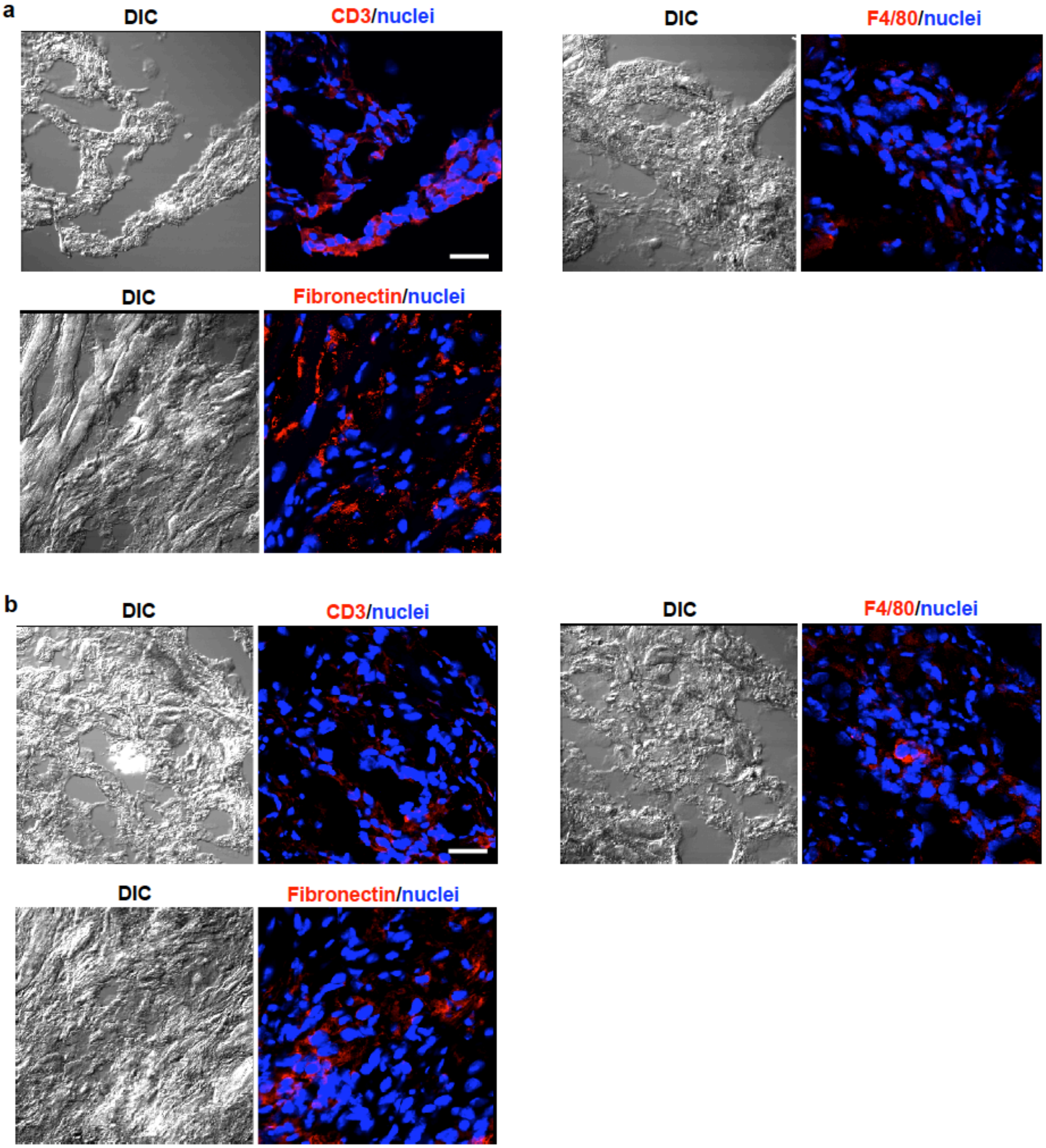

Supplementary Figure 7. Typical micrographs of the granuloma tissue with immunofluorescence staining. **a**, granulomas harvested from single cell treated wild type (WT) mice. **b**, granulomas harvested from bare-A treated WT mice. All granulomas were collected at 28 days after injection. All the tissue sections are stained positive for macrophages (F4/80) and T cells (CD3), as well as fibroblasts (fibronectin), which is typical for granuloma. Scale bar: 20 µm

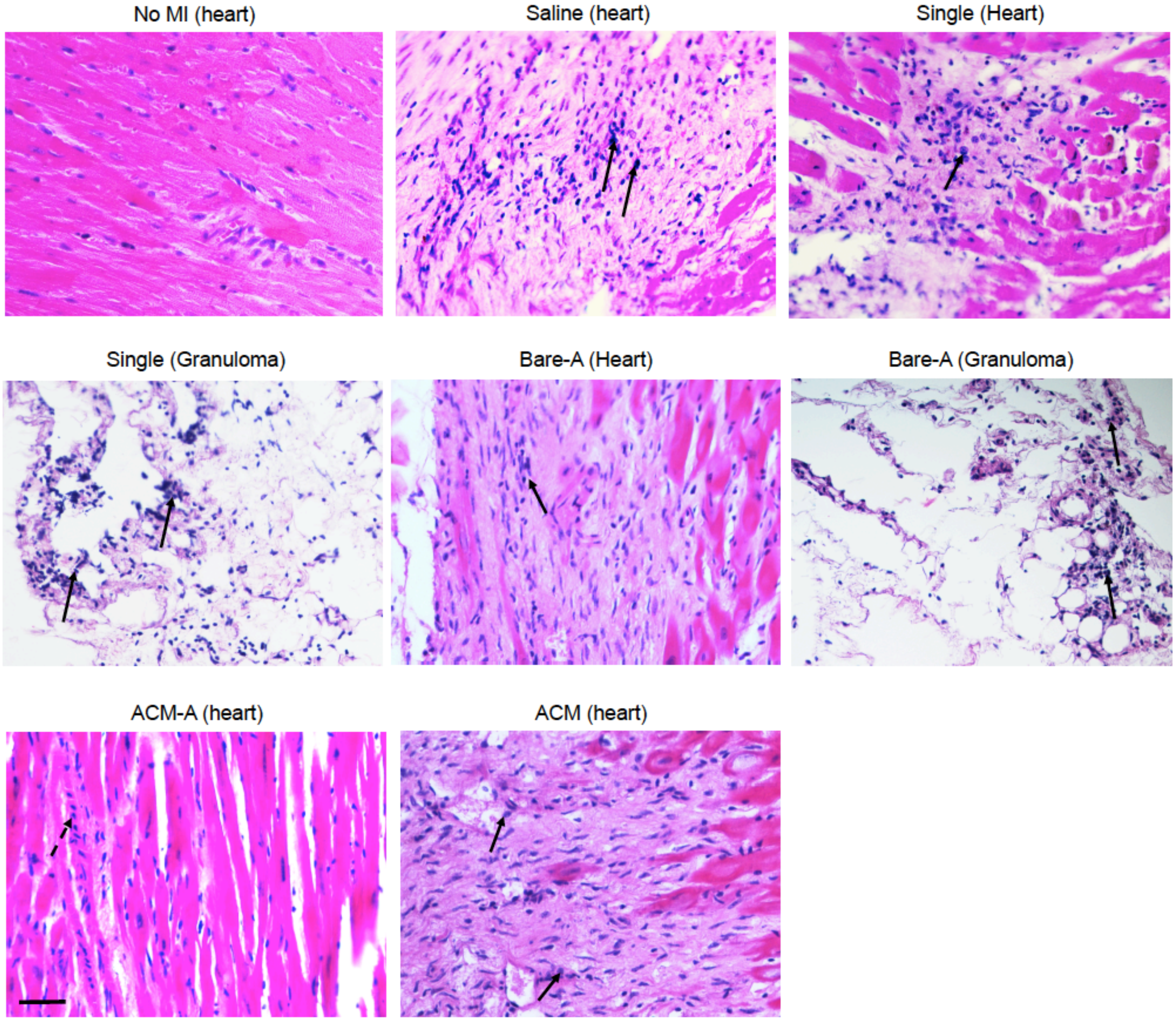

Supplementary Figure 8. High magnification images showing histology of heart tissues and granulomas. The tissues were stained by hematoxylin and eosin (H&E). In the MI heart treated with saline, single cells (Single), Bare-A, and ACM, there are macrophages (solid arrows) and fibroblasts in the fibrotic area, while fibrosis is negligible for the No MI control and minimal in the ACM-A treated heart. There are some degenerated cardiomyocytes with multiple nuclei (dashed arrow) in the ACM-A treated hearts, but no massive macrophages. In addition, the granulomas from single cells and bare-A treated mice exhibit strong inflammation indicated by the presence of massive macrophages (arrows). Scale bar: 50 μm

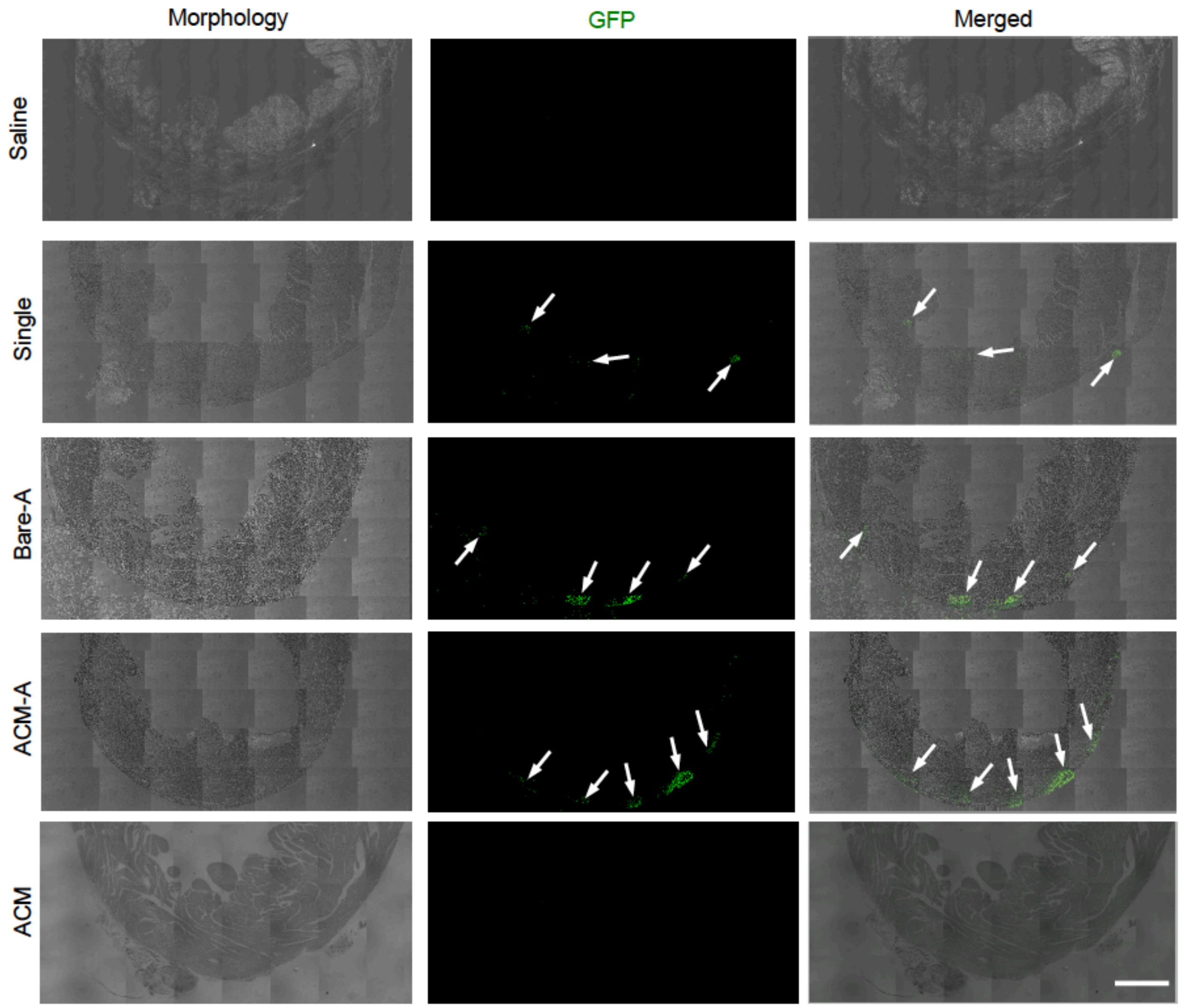

Supplementary Figure 9. Retention of implanted cells in the left ventricular wall. Low magnification phase and fluorescence micrographs and their merged views of tissue sections from the left ventricle of MI heart of wild-type mice at 28 days after treated with saline, single cell (Single), Bare-A, ACM-A, and ACM. The implanted cells with green fluorescence are visible for all the groups with cells (Single, Bare-A, and ACM-A) and the ACM-A treatment leads to the highest cell retention. No green fluorescence was observed in the treatment groups without cells (Saline and ACM), indicating the green fluorescence is due to the implanted cells with green fluorescent protein (GFP) rather than auto fluorescence of scar tissue that is must abundant in the saline and ACM treated heart. Scale bar: 1 mm. The images were produced automatically using Zeiss MosaiX for tiling and imaging stitching.

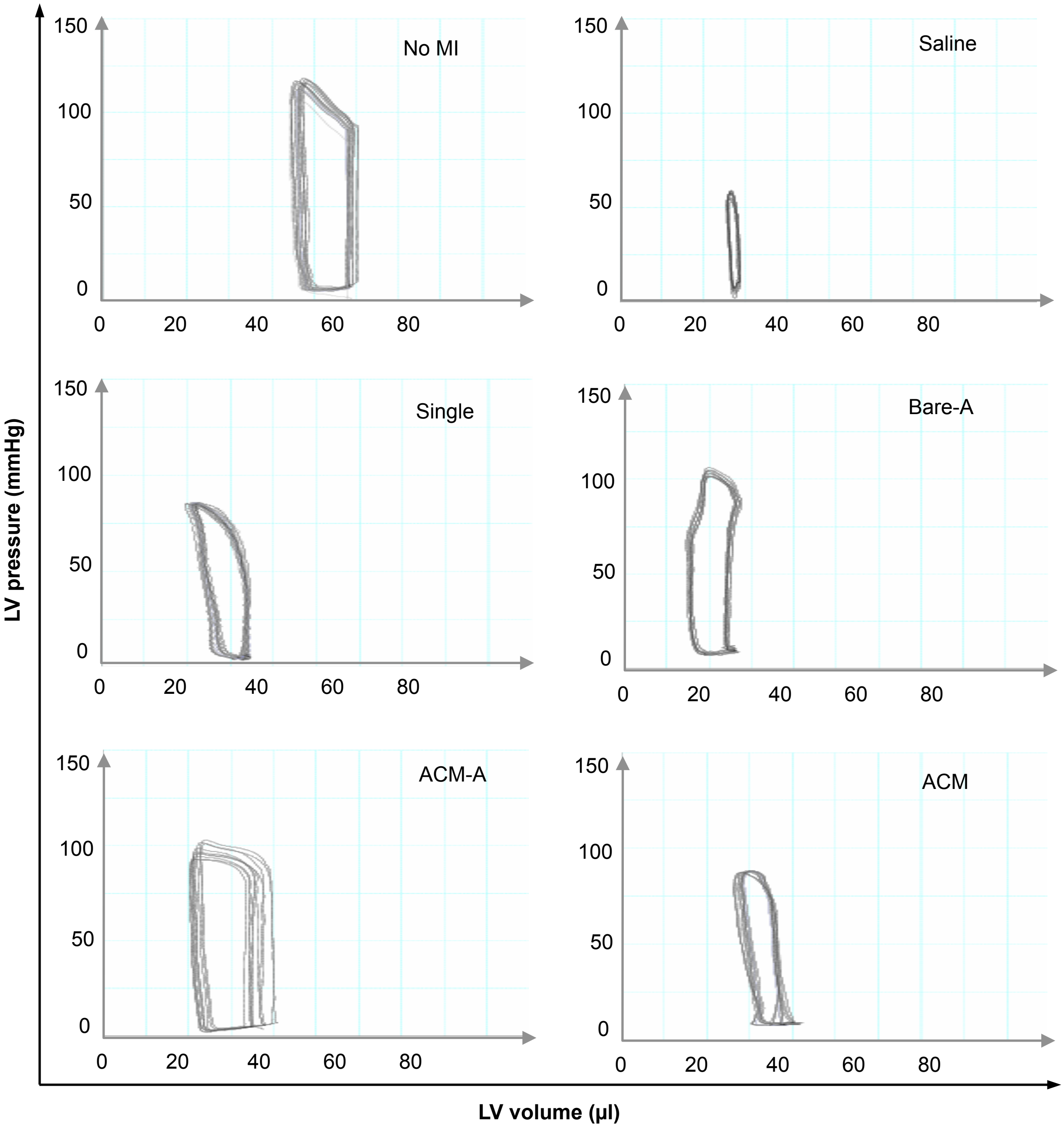

Supplementary Figure 10. **Typical micrographs of left ventricle pressure versus volume of mice.** The data were collected at 28 days after surgery. The left ventricle (LV) pressure-volume (PV) loop data indicate that severely reduced heart function of saline treated MI mice, limited improvement of heart function for the single cell, Bare-A, and material alone (ACM) treatments, and evident improvement of heart function with the ACM-A treatment.

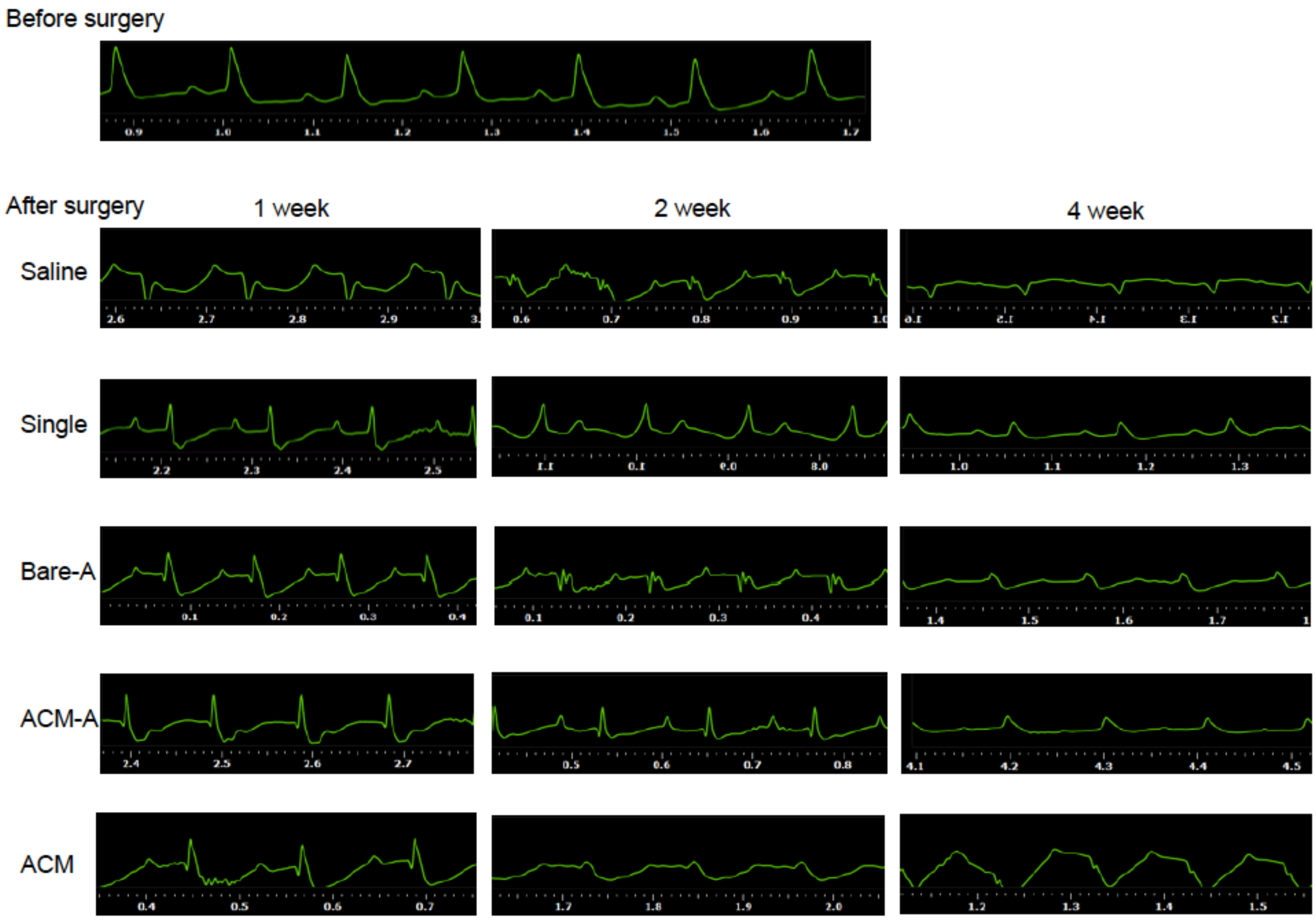

Supplementary Figure 11. **Typical electrocardiograms of mice before and after surgery.** During the first week, all the mice with MI show marked ST-segment elevation, particularly for the saline-treated mice that have inverted QRS and T. In the second week, ST-segment of Single, ACM-A, and Bare-A groups gradually recovered, while mice treated with saline and ACM still had ST-segment elevation. At 4 weeks, although all groups showed enlarged QRS and flattened T, the groups with cells are less severe than the saline and ACM groups that also have inverted QRS. In addition, no evident arrhythmia is observable for any of the treatments.

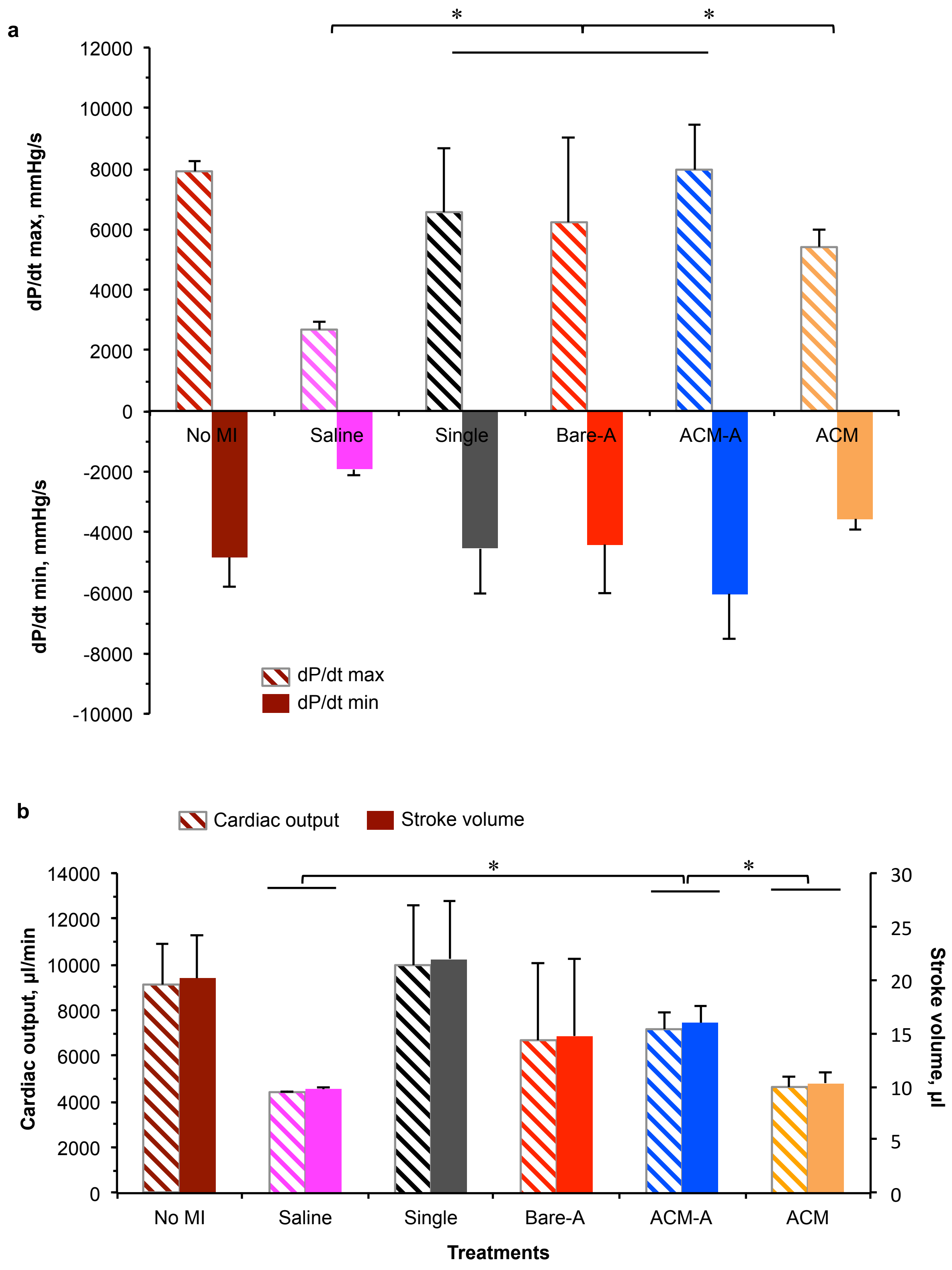

Supplementary Figure 12. Cardiac functional data from PV loop measurements at 28 days after surgery. a, Maximum and minimum rate of pressure change (dP/dt). **b**, Cardiac output and stroke volume. The data show that the treatments with cells (Single, Bare-A, and ACM-A) significantly improve the heart function of mice with MI, compared to the saline and ACM treatments with no cell. Error bars represent s.d. (n=4 for Single and n=3 for all other groups). *, $p < 0.05$ (one-way ANOVA)

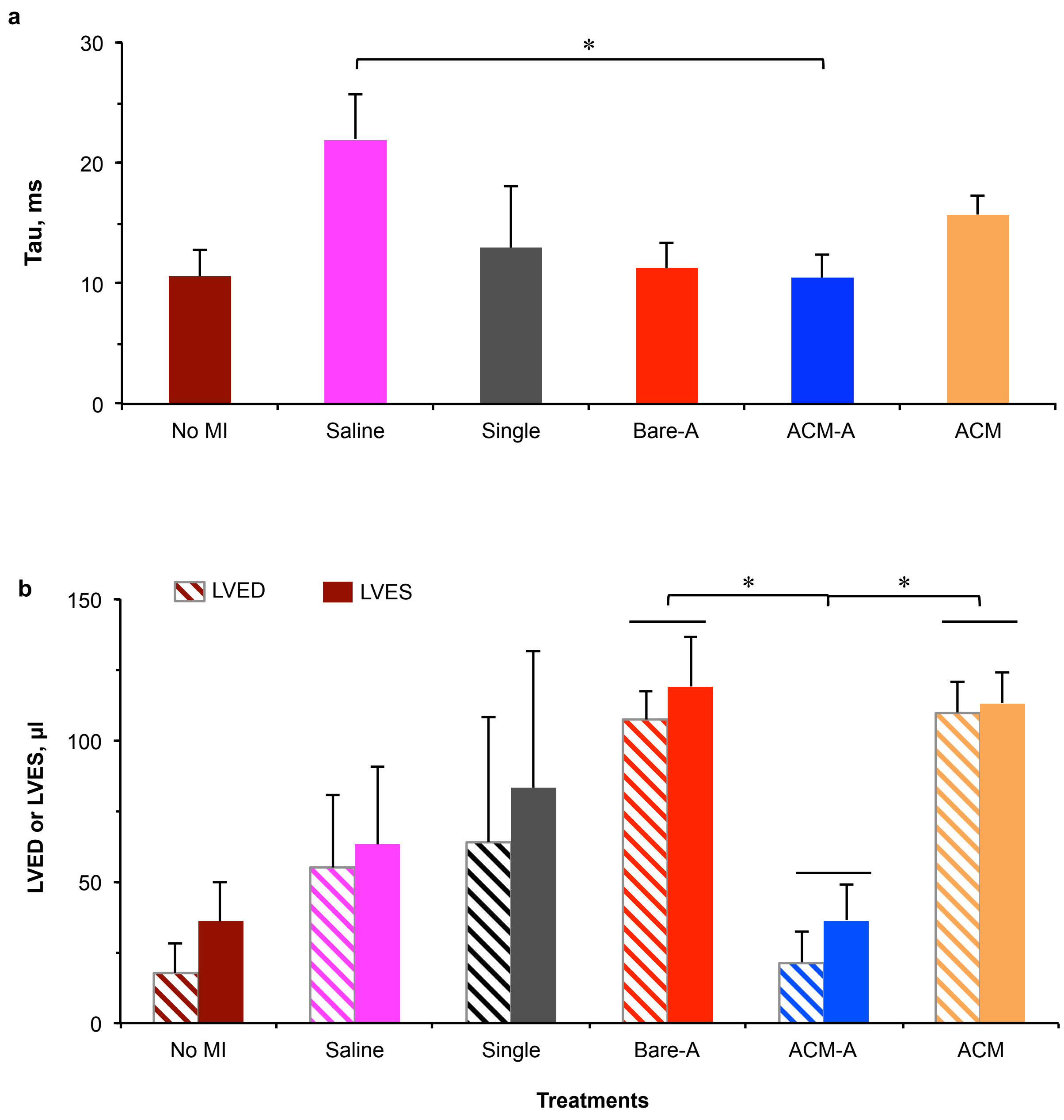

Supplementary Figure 13. Cardiac functional data from PV loop measurements at 28 days after surgery. a, Isovolumic relaxation constant (Tau). **b**, Left ventricle (LV) end-diastolic volume (LVED), and LV end-systolic volume (LVES). The data indicates ACM-A improves heart function by consistently maintaining a nearly intact isovolumic relaxation with minimal enlargement of the left ventricle (LV), compared to the other treatments. Error bars represent s.d. (n=4 for Single and n= 3 for all other groups). *, $p < 0.05$ (one-way ANOVA)

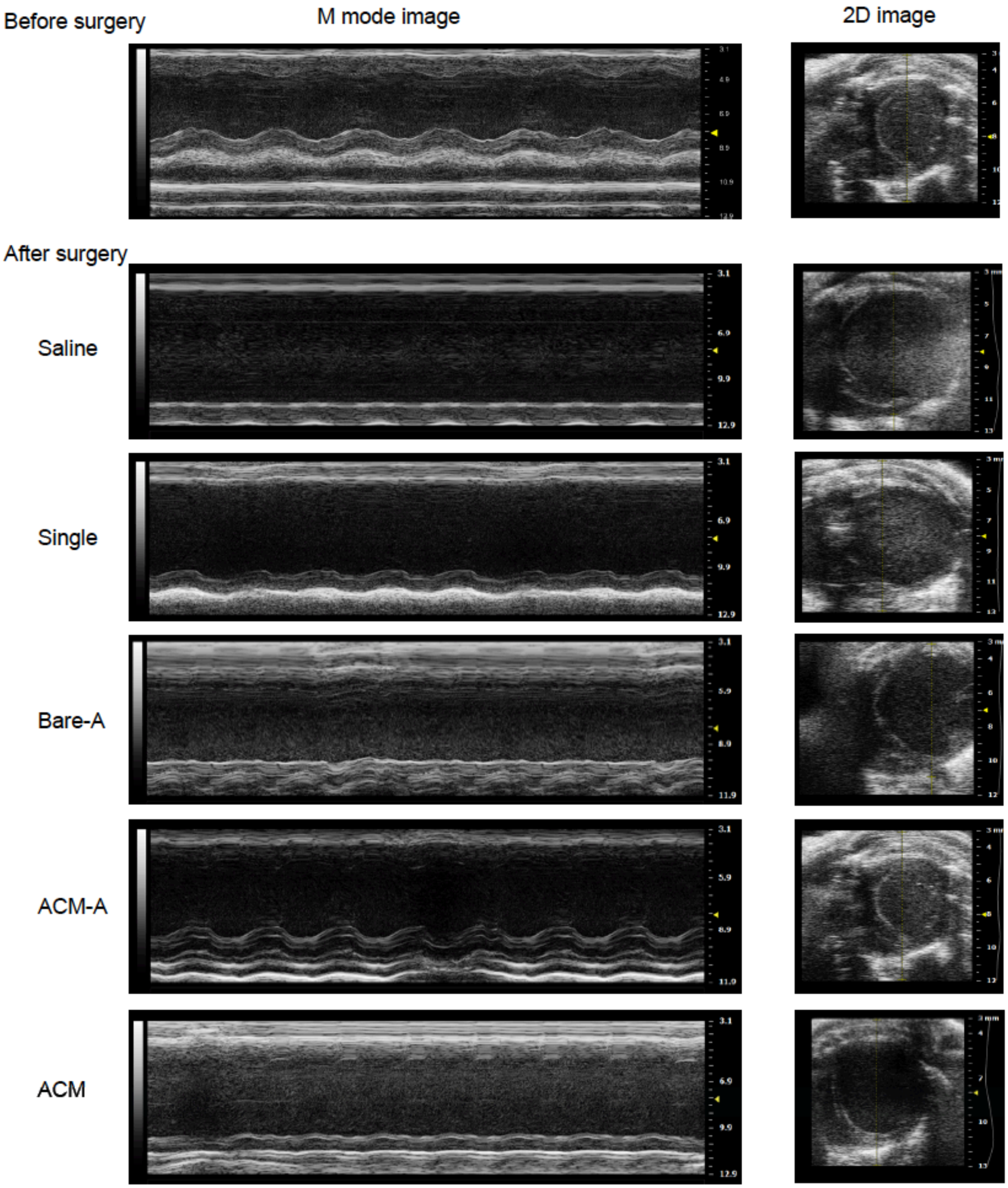

Supplementary Figure 14. **Representative echocardiograms of mice before and at four weeks after surgery.** All mice were under anesthesia during the echocardiography experiments. By four weeks, the MI mice in all treatment groups show major changes in heart structure while the change is the least for mice with the ACM-A treatment. The ventricular contraction amplitude for the ACM-A treatment is improved compared to that for the other four treatments.

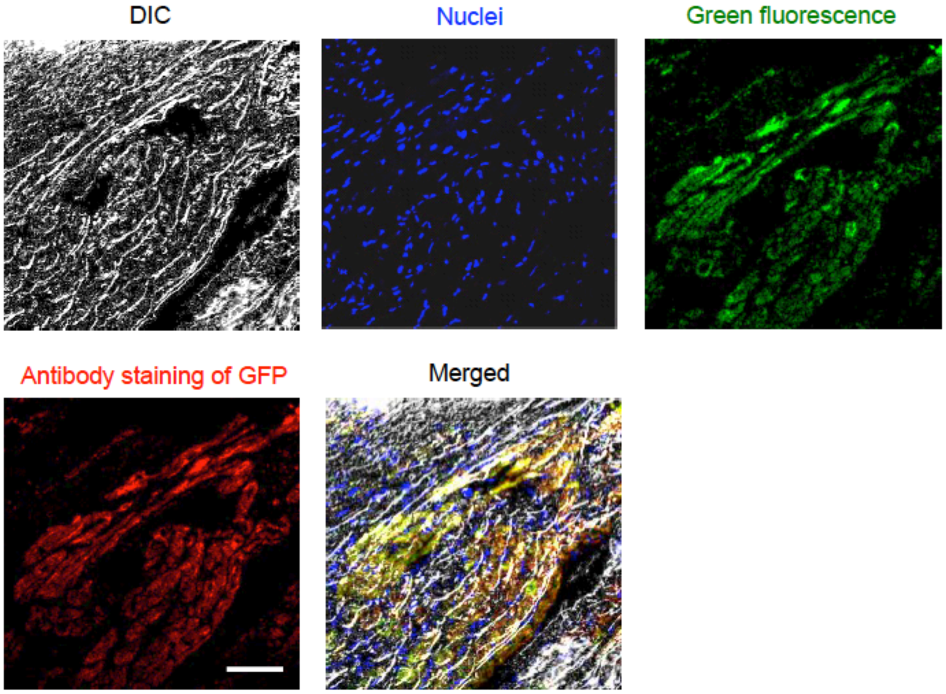

Supplementary Figure 15. Antibody staining of green fluorescent protein. The green fluorescent protein (GFP) was stained in tissue from the infarct zone of heart treated with ACM-A made of GPF positive cells. The extensive co-localization of the green fluorescence with the antibody staining of GFP (in red) indicates the green fluorescence is indeed from the implanted cells with GFP expression. The cell nuclei were stained with DAPI (blue). DIC: differential interference contrast. Scale bar: 100 μm.

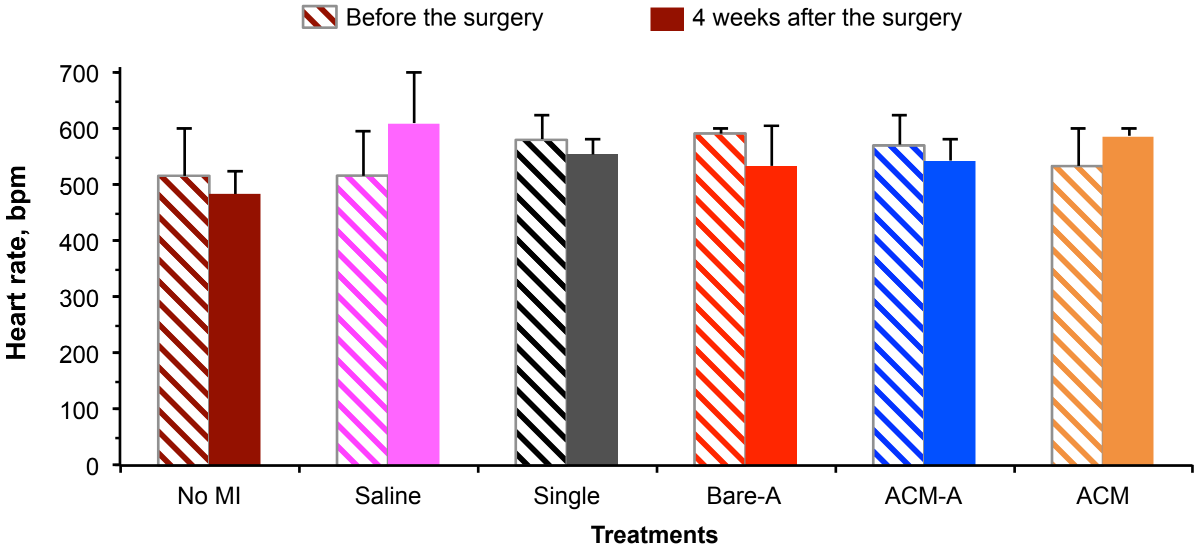

Supplementary Figure 16. Heart rate measured by echocardiography before and after surgery. It shows no significant difference among the different treatment groups before surgery (baseline) and at 4 weeks after the surgery (endpoint). Error bars represent s.d. The n=4, 9, 8, 5, 8, and 4 for No MI, Saline, Single, Bare-A, ACM-A, and ACM, respectively. No significance was detected between the different groups (one way ANOVA).

Supplementary Table 1. A list of the number of animals used for all *in vivo* studies. At least 3 mice were used for each experimental group for all the *in vivo* studies.

Studies	Groups	Number (n)	Studies	Groups	Number (n)
**Animal survival**	Saline	31	**Cell survival**	Saline	3
	Single	29		Single	3
	Bare-A	38		Bare-A	3
	ACM-A	31		ACM-A	3
	ACM	24		ACM	3
**Histology/fibrosis**	No MI	3	**PV loops**	No MI	3
	Saline	3		Saline	3
	Single	3		Single	4
	Bare-A	3		Bare-A	3
	ACM-A	3		ACM-A	3
	ACM	3		ACM	3
**Immunostaining**	No MI	3	**Echocardiography**	No MI	4
	Saline	3		Saline	9
	Single	3		Single	8
	Bare-A	3		Bare-A	5
	ACM-A	3		ACM-A	8
	ACM	3		ACM	4
**Granuloma observation**	No MI	10			
	Saline	31			
	Single	29			
	Bare-A	38			
	ACM-A	31			
	ACM	24			
	Bare-A-KO	9			
